# Annual migrations, vertical habitat use and fidelity of Atlantic bluefin tuna tracked from waters off the United Kingdom

**DOI:** 10.1038/s41598-024-80861-w

**Published:** 2025-01-02

**Authors:** Thomas W. Horton, Francis C. T. Binney, Samantha Birch, Barbara A. Block, Owen M. Exeter, Francesco Garzon, Alex Plaster, David Righton, Jeroen van der Kooij, Matthew J. Witt, Lucy A. Hawkes

**Affiliations:** 1 Environment and Sustainability Institute, University of Exeter, Penryn, TR10 9FE UK; 2 Hatherly Laboratories, University of Exeter, Prince of Wales Road, Exeter, EX4 4PS UK; 3 Centre for Ecology and Conservation, University of Exeter, Penryn Campus, Penryn, Cornwall, TR10 9FE UK; 4 Government of Jersey Marine Resources, Natural Environment, Howard Davis Farm, Trinity, Jersey; 5 Centre for Environment, Fisheries and Aquaculture Science, Pakefield Road, Lowestoft, NR33 0HT UK; 6 Department of Oceans, Stanford University, Hopkins Marine Station, Pacific Grove, CA USA; 7 School of Environmental Sciences, University of East Anglia, Norwich, NR4 7TJ UK

**Keywords:** Animal behaviour, Zoology, Marine biology, Ecology, Animal migration, Conservation biology

## Abstract

Understanding the spatial ecology of commercially exploited species is vital for their conservation. Atlantic bluefin tuna (*Thunnus thynnus*, ABT) are increasingly observed in northeast Atlantic waters, yet knowledge of these individuals’ spatial ecology remains limited. We investigate the horizontal and vertical habitat use of ABT (158 to 241 cm curved fork length; CFL) tracked from waters off the United Kingdom (UK) using pop-up satellite archival tags (*n* = 63). Analyses reveal distinctive movements from the UK to the Bay of Biscay (BoB) and Central North Atlantic between September and December, and size-specific habitat preferences in May and July—all ABT < 175 cm CFL inhabiting the BoB and 73% of ABT ≥ 175 the Mediterranean Sea. All ABT tracked for more than 300 days (*n* = 25) returned to waters off the UK the following year, where most stayed (*n* = 22; 88%) and three continuing north with deployments ending off northwest Ireland. ABT mostly occupied waters between 0 and 20 m (daytime 49 ± 6% of time; nighttime 71 ± 6%). Vertical habitat use was coupled with illumination, mean depth occupied, maximum depth reached, and vertical movement rate increased during the daytime and when moons were brightest. These data provide valuable insights into the spatial ecology of ABT reoccupying northerly foraging areas following decades of absence.

**Supplementary Information:**

The online version contains supplementary material available at 10.1038/s41598-024-80861-w.

## Introduction

The Atlantic bluefin tuna (ABT, *Thunnus thynnus*) is a highly migratory fish that occurs across the North Atlantic Ocean^1^. ABT command a high commercial value, with populations historically overfished as a result^2,3^. Fisheries for ABT are managed by the International Commission for the Conservation for Atlantic Tuna (ICCAT), which consider the ABT population as two spatially discrete stocks, eastern and western, separated by the 45° W meridian, with structure maintained by natal homing^4^. The eastern stock is significantly larger than the western stock and has grown over the last decade to a relatively high biomass^5^, while the western stock is yet to recover from overfishing^6^. Recovery of the eastern stock has coincided with the return of ABT to northern waters off the United Kingdom (“UK”^7^), the Channel Islands archipelago (“CI”: Jersey, Guernsey, Alderney and Sark, which are British Crown dependencies^8^), Norway^9^, Denmark and Sweden^10^, as well as first recorded occurrences in the waters off Greenland^11^, and increased occurrence in the western Atlantic^12^. The return of ABT to these areas likely reflects re-occupation of their historical range through fluctuations in climate and/or the availability of prey species and preferred habitat^13–15^.

Understanding the movements of different sub-populations and how they mix across their range and throughout various life stages is of direct importance for management of the ABT fishery by ICCAT. Additionally, close-kin mark-recapture stock assessment methods under consideration by ICCAT^16^ can provide valuable insights into population structure and connectivity^17^ and can be significantly enhanced by information on movement patterns. Despite this, data on the portion of the ABT population that seasonally occupy the shallow Channel between southern UK and northern France (from hereon, “the Channel”) are limited. For example, individuals have never been tracked from the region (e.g^1,18–20^), only one has been tracked to the region^21^ and there has not been a fishery in the UK since ICCAT was formed in the 1960s^22^. Therefore, the return of ABT to waters of the Channel represents a knowledge gap, and studying the movements of ABT that now reside seasonally in the region will provide information pertinent to their management and conservation.

Studies using electronic tags in other parts of the species’ range have provided vital insights into the movements, spatial ecology, and population structure of ABT^1,18,19,23–25^, including important areas^18,25^ and behaviours indicative of either foraging^26–28^ or spawning^20,29^. In this study we used pop-up satellite archival tags (PSATs) to study the movements and habitat use of ABT tracked from foraging aggregations in the Channel between 2018 and 2021. The specific aims of this study are to: (1) provide an overview of the general movements and habitat use of ABT present in the recently re-established foraging grounds in the Channel, and (2) consider these new data in the context of their spatial ecology and conservation, and the broader population (e.g. stock of origin, spawning ground usage, connectivity with other known aggregation sites and key life history traits). The overarching goal is to expand the knowledgebase concerning the recent distributional change of this ecologically important apex predator.

## Materials and methods

### Ethics

All research was conducted under UK Home Office license (Project licenses P23C6EFD2 and P9D31EA7F) and was reviewed by the University of Exeter and Cefas Animal Welfare and Ethical Review Boards (AWERBs). Scientific fishing for Atlantic bluefin tuna was conducted under fisheries dispensations from the Marine Management Organization (037/18, 23/19, 14/20 and 14/2021), the Welsh Government (DISP089) and the Government of Jersey (CR98). All authors complied with ARRIVE guidelines.

### Electronic tagging

ABT (*n* = 63; ranging from 153 to 242 cm CFL, mean ± 1 S.D. = 198 ± 24 cm) were captured in waters off England (*n* = 55), Wales (*n* = 3), and the Channel Islands (*n* = 5) between 2018 and 2021 by licenced vessels and anglers using heavy tackle, either with trolled lures and J-hooks or dead baits with circle hooks, and brought to the boat as quickly as possible. Tagging was conducted using established techniques^23^ refined for regional vessels and legislation. ABT were boarded using a lip hook to pull the fish through a transom or side door onto a wet padded vinyl mat, and the eyes were covered using a clean microfibre towel covered in fish slime protector solution (Fish Protector™, Kordon). An ambient temperature saltwater hose was used to irrigate the gills (flow rates approximately ~ 10 to 18 l min^− 1^). PSATs were secured externally using a 15 cm tether anchored into the dorsal musculature by a custom-manufactured titanium dart. A second tether (“loop”) was placed around the tag trunk and also inserted 15 cm into the base of the second dorsal musculature to prevent lateral movement of the TAs^23^. On board the vessel, each fish was measured (half girth and curved fork length, CFL), a fin clip and a muscle biopsy were taken (for genetic identification), and a plastic ICCAT identification tag (Floy; Washington, US) was inserted into the dorsal musculature on the opposite flank to the electronic tag. PSATs (“tags” hereafter) used were Wildlife Computers MiniPATs (model 347 and 348) programmed for deployments of 330 to 365 days (*n* = 54) or 730 days (*n* = 9). Tags archived data (i.e. depth, temperature, light, and acceleration) at either 5 (*n* = 43) or 15 s frequency (*n* = 20), which could be directly downloaded if the tags were physically recovered. All tags were programmed to detach if they were either floating at the surface or remained at a constant depth (± 2.5 m) for three days, or if the tag was deeper than 1400 or 1700 m dependant on tag firmware. Most (*n* = 62 tags) tags had a low-power, high-frequency (2-s) “pinger” activated to aid physical recovery. All tags were programmed to transmit daily light curves, sea surface temperature (SST; temperature within the top 5 m of the water column) and maximum dive depths for geolocation. Tags were programmed following manufacturer guidelines to generate a maximum of 1,800 (pre 2020) or 1,400 (2020 onwards) data messages to transmit. If, after pop-up, tags were either floating near the coast, or beached on accessible coastline, they were recovered using an R20 or R30 (iCOM UK Ltd.) with directional antenna and an ARGOS Goniometer CLS RXG-234 (CLS, France).

### Geolocation and dataset preparation

Movements of tagged animals were reconstructed using light based geolocation^30^ software, the Global Position Estimator 3 (GPE3). Wildlife Computers’ GPE3 is a space-time discretised Hidden Markov model that uses tag derived observations of ambient light (daily light curves), SST and maximum daily depth^31,32^. These data are cross referenced with NOAA high resolution SST (http://www.esrl.noaa.gov/psd) and the ETOPO1 bedrock bathymetric model^33^. Location estimations are constrained at the beginning of the tracking using deployment locations (from hand held GPS) and at the end using ARGOS satellite geolocation endpoints (the first class 1 to 3 location after reporting, with associated error of 400 m or less^34^). The user defined movement speed used for all track reconstructions in GPE3 was 2.5 m s^− 1^, which we derived empirically from earlier work in Horton et al.^35^. Briefly, using the GPE3 we processed a representative subset of tags (attached to ABT with CFLs between 158 and 238 cm; *n* = 11) at movement speeds from 0.5 to 5 m s^− 1^, at 0.5 m s^− 1^ increments (i.e. one GPE3 run per increment per tag). We then used the GPE3 score as an indicator of goodness of fit and plotted data to identify an asymptote (Fig. S1) that would inform our global movement speed. Our analysis revealed that this asymptote was reached at 2.5 m s^− 1^ and does not appear to vary with the CFL of the fish the tag was attached to indicating suitability for our entire dataset. In addition, tracks that were derived from less than 30% of total geolocation data were excluded from analyses (also following Horton et al.^35^).

Movements reconstructed by the GPE3 provide multiple locations per day, which were averaged (geodesic mean) to provide single daily location estimates for each tag. One individual was presumed to have died 3 h after release (remained at a constant depth consistent with that of the seabed for three days) and was removed from the final dataset. Geolocation and diving data for one individual indicated it may have been caught in a trap between the 23rd of May and the 28th of June, before being released (Fig. S2). Due to this ambiguity, data from this fish over this period were omitted from spatial analyses for this tag. Depth sensors on six tags malfunctioned over a portion of the deployment, and these segments of data were omitted from diving analyses. For these tags, the temperature readings at the minimum daily depth for the sensor affected days were deemed to constitute SST (under the assumption that ABT occupy near surface waters every day of tracking^35^), and were incorporated in GPE3 processing. Downloaded archives from recovered tags were down sampled from 5 to 15 s, where necessary, to match the lowest sampling frequency for all deployments.

### Analyses

#### Horizontal movements

**Fig. 1:**
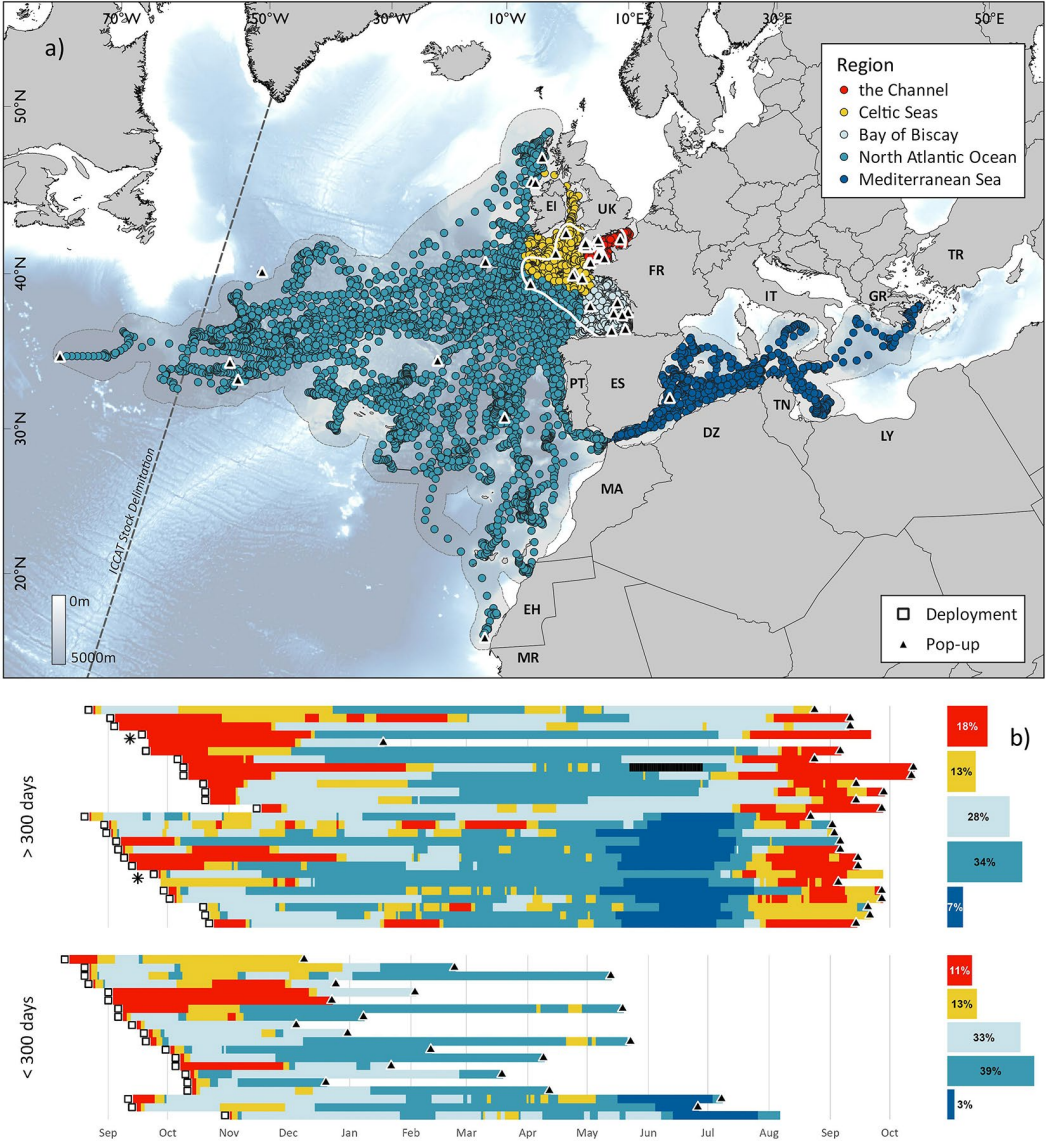
Dispersal behaviour of 45 ABT tracked from the United Kingdom and Channel Islands. (**a**) Modelled daily locations for 45 ABT. Grey shaded area denotes the 99% probability surface for all tags combined. White contour represents the greatest density of points comprising 60% of all locations (*n* = 8100) approximated using kernel density estimation. (**b**) Residency of individual ABT (one per row) over time summarised by IHO region occupied. Inset barplot shows grand mean proportion of time in each IHO region as a percentage. Black denotes a period where an individual was considered to be temporarily caught in a tuna trap (Fig. S1). EEZs that ABT were tracked in are labelled with two letter codes. Map created in QGIS v3.16^39^.

Analyses and mapping were conducted in R^36^ using RStudio^37^ and additional mapping in Fig. 1 using QGIS^38^. Location uncertainty was calculated using the 99% probability contour of each 12-hourly probability raster (0.25 × 0.25° grids) provided by the GPE3 model. For all tags that remained attached for 300 days or more, the grand latitudinal mean uncertainty was 1.18 ± 0.25° and the grand longitudinal mean uncertainty was 1.63 ± 0.35°.

Shapefiles of Exclusive Economic Zones (EEZs) and International Hydrographic Organization (IHO) Sea Regions were obtained from http://www.marineregions.org/downloads and simplified to suit study aims (Fig. S3). Rasters of 12-h occurrence probability (as above) were used to compute the probability of tags occurring within each EEZ, and within each simplified IHO region by each of three temporal phases. For tags remaining attached for 300 days or more (*n* = 25), probabilities of occurrence were calculated for each tag for each phase (e.g. August to November) by summing all probabilities within an EEZ/IHO combination and dividing by the total summed probability for that tracking phase. Grand mean seasonal probabilities were then calculated from seasonal tag means. For visualisation, the summarised seasonal probability rasters were overlaid and combined by summing using the “terra” package in R^39^, before normalising by dividing by the sum of all raster cells (Table S2). As a supplementary analysis, we also calculated the estimated time (as number of days—Table S3; and, as the percentage of a summary period—Table S4) ABT spent within each EEZ/IHO combination.

Body size has been shown to affect ABT distribution and migratory behaviour into and out of the Mediterranean Sea^1,21^. To investigate the body size of ABT entering the Mediterranean Sea (“Med.”), the CFL of fish at the date they first conducted a west-east crossing through the Strait of Gibraltar at 5° W was estimated. This was done by using the size at release, applying daily growth equations from Cort et al.^40^ and using conversion factors from Rodriguez-Marin et al.^41^. The relationship between body length of ABT and entry to the Med. was investigated using a non-parametric Wilcoxon rank sum test on two samples due to the differences in sample sizes.

#### Vertical habitat use

To investigate vertical habitat use, we analysed high resolution (15 s interval) data downloaded from recovered tags. Sunrise and sunset times derived from light levels (via GPE3 outputs) were used to partition archival tag data into either daytime or nighttime periods. For each daytime or nighttime period we calculated three metrics to describe vertical habitat use: mean depth occupied (mean depth), maximum depth reached (maximum depth), and vertical movement rate (VMR the absolute depth change in meters divided by the length of the summary period in minutes). Prior to modelling, covariance between paired metrics (i.e. mean depth *versus* maximum depth) was assessed using Spearman’s rank order correlations. Due to all three metrics being positively correlated at the P = ≤ 0.001 level (ρ values given in Fig. S9), we investigated each metric independently. Gaussian Generalized Linear Mixed Models (GLMM) were fitted to either root (for VMR) or log transformed (for mean depth and maximum depth) data using the *lme* function from the “nlme” package in R^42^. A model was constructed with each of mean depth, maximum depth and VMR as responses. Fixed effects for models included: (i) day or nighttime, (ii) lunar illumination, (iii) horizontal displacement, (iv) sea region (as above), and, (v) phase (on-shelf, off-shelf and the spawning season) while inter-individual variation was accounted for by specifying tag ID as a random effect. To address temporal autocorrelation in residuals, we incrementally updated the null model with six different autoregressive-moving average (ARMA) structures using the *corARMA* function in the “nlme” package^42^ (Table S5). Model fit was assessed by comparing each updated model to the null model using the *model.sel* function from the “MuMIn” package^43^. We then applied the Ljung-Box test on standardized residuals with *Box.test* function from the “stats” package in R^36^, where a non-significant result indicated no remaining autocorrelation^44^. The final model retained the simplest ARMA structure that met this criterion, balancing accuracy with parsimony. Lunar illumination was extracted for each discrete time point using the *getMoonIllumination* function in the “suncalc” package^45^. Horizontal displacement (km.day^− 1^) was calculated as the distance between successive daily locations. Interaction terms were specified for daytime period and moon illumination and sea region and phase. For the latter, interactions were specified manually to avoid model singularities arising due to the Mediterranean Sea only being occupied during the spawning season. Final models were checked by visually inspecting standardized residuals using the *check_model* function in the “Performance” package^46^. The significance of individual fixed effects was assessed using the *drop1* function in R, by sequentially dropping each fixed effect and comparing the reduced model compared with the full model using a Likelihood Ratio Test. Significance was determined by comparing the likelihood ratio test statistic to a chi-squared distribution with degrees of freedom (calculated using Satterthwaite’s method;^47^) equal to the difference in the number of parameters between the full and reduced models. To investigate within group relationships, Tukey-adjusted pairwise comparisons of estimated marginal means were conducted using the *emmeans* function in the “emmeans” package^48^. For all statistical tests, the significance threshold was set at *P* ≤ 0.05. Unless stated otherwise, means are provided ± 1 standard deviation.

## Results

### Tagging

Data were received from 56 tags (89% of 63), which collected data over 1,471 consecutive days between the 3rd October 2018 and the 13th October 2022. Of these, ten tags (16%) transmitted less than 30% of geolocation data (i.e. insufficient data for reliable movement reconstructions^49^, and one fish died post release. Following removal of these tags from the study, remaining tags (*n* = 45; ABT size 153 to 242 cm CFL) were attached for 282 ± 125 days (range 67–708 days) and collected 12,738 cumulative days of data. In total, 25 ABT were tracked for 300 days or more (i.e. a complete, or near complete, annual migratory cycle) and 25 tags were physically recovered (41% of 63 tags), and data downloaded (resulting in 8,546 cumulative days of archival data).

### Annual migratory cycle

**Fig. 2:**
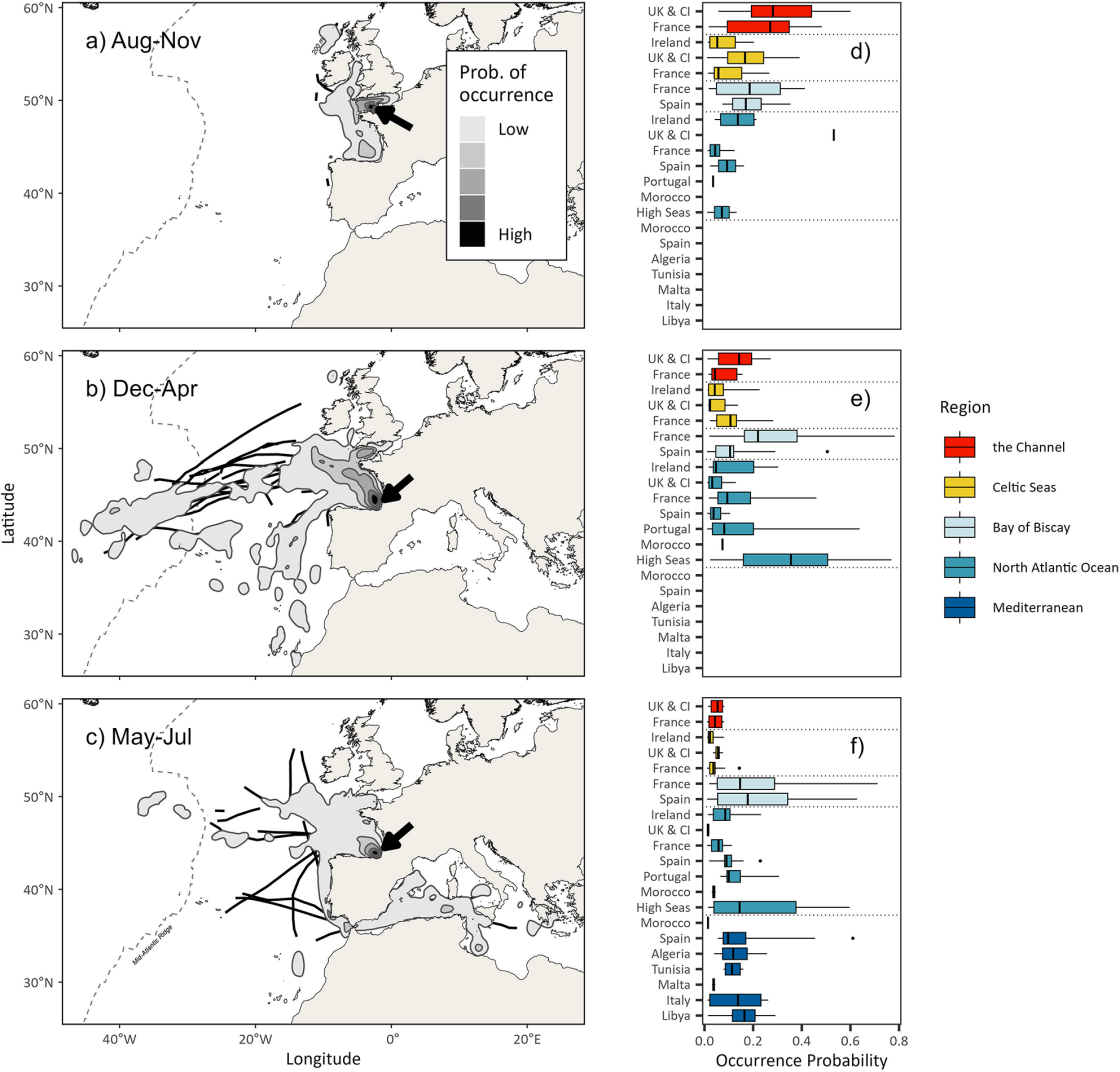
Seasonal movements and space use of ABT that were tracked for more than 300 days. (**a**–**c**) Rasters of mean occurrence probability for 25 ABT in three periods of the year. Black lines denote periods of 7 days or more where swimming speeds were sustained above 90 km per day. Arrows on each plot indicate locations where ABT were most likely to be found. (**d**–**f**) Boxplots of occurrence probability for individual ABT occurring within exclusive economic zones (EEZs) and IHO regions defined in Fig. 1. Map created using R^37^ and RStudio^38^.

Tags (*n* = 45; Table S1) revealed movements throughout the northeast Atlantic: west to the Central Atlantic Ocean (56°W), north to Scotland, south as far as the Canary Islands and to the east as far as Turkey in the Med. (Fig. 1a,b). For all ABT pooled, 99.4% of locations occurred in the eastern stock management region (*n* = 12,662 days). Tags attached for 300 days or more revealed annual migrations comprising three phases (Fig. 2a–c): an on-shelf phase between August and November where ABT remained in coastal waters on the northwest European shelf; 2) an off-shelf phase between December and April whereby a proportion of ABT (*n* = 11, 44%) migrated to High Seas waters (but 56% remained near to the shelf; Fig. S6), and 3) the spawning season between May and July, where ABT either migrated to known spawning regions in the Med. (*n* = 13) or remained in south-east Bay of Biscay (“BoB”; *n* = 12). Throughout the year, ABT resided in 11 EEZs (Fig. 2d–f). During the on-shelf phase, individuals were most likely to occupy French or UK & CI EEZs in the Channel (grand mean probability of occurrence in French EEZ was  0.23 ± 0.14, and in the UK & CI EEZ was 0.32 ± 0.18; Table S2). In the Channel and Celtic Seas regions, highest location densities occurred west of the CI and north of Brittany, France (Fig. 2a).

**Fig. 3:**
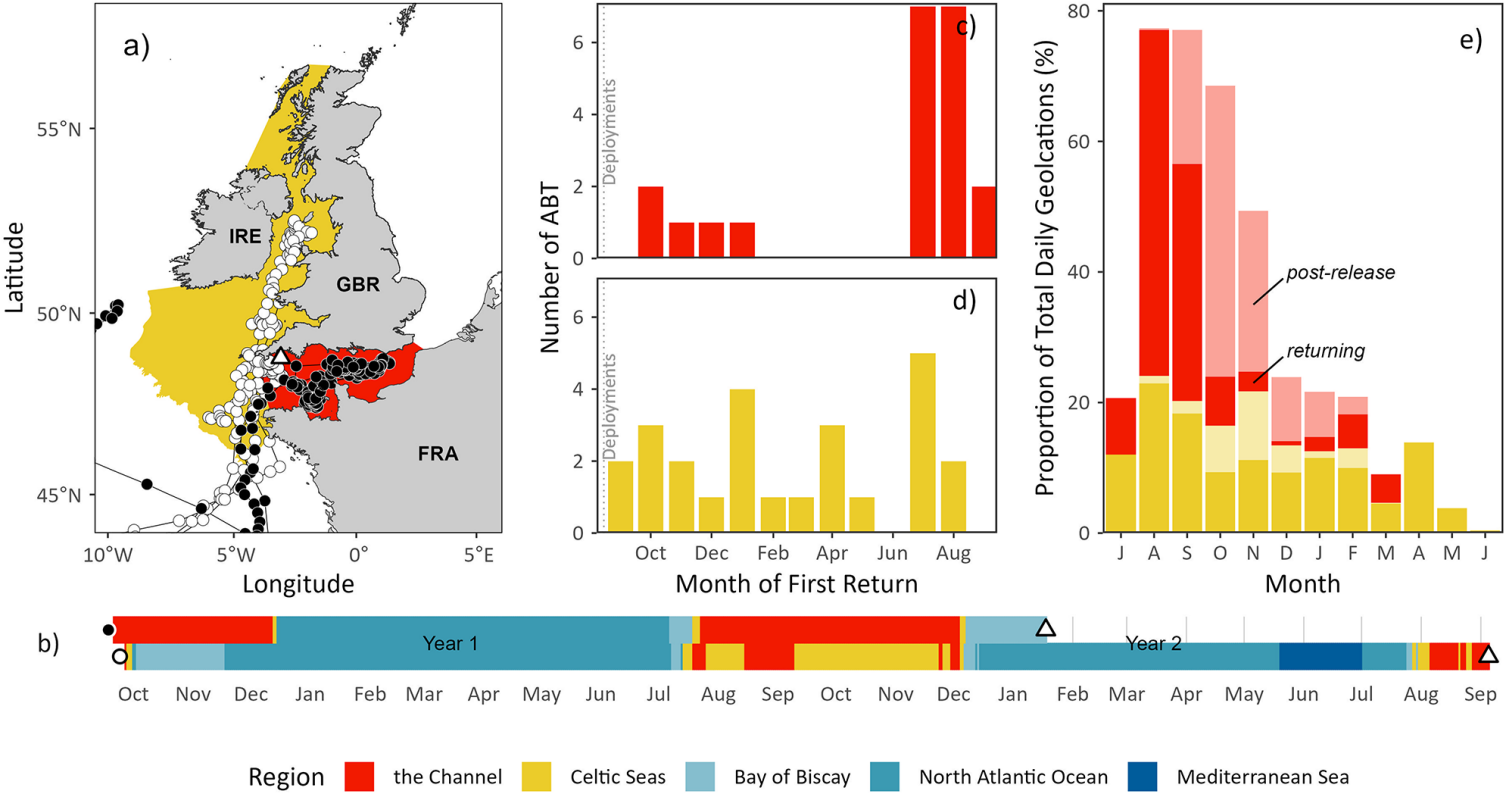
Phenology of ABT on the north-western European shelf. (**a**) Modelled daily locations in the northeast Atlantic for two ABT tracked for 481 (black) and 708 days (white), with movements of individual ABT joined by lines, overlaid on coloured polygons denoting simplified IHO regions used in this study. (**b**) Residency of ABT depicted in “(**a**)” (one per row) over time summarised by IHO region occupied. (**c**,**d**) Timings of ABT returning (i.e. having left post-release) to each of the two regions shown on map. Vertical dotted line denotes the mean deployment month. (**e**) Monthly proportion of daily geolocations in regions shown in “(**a**)”, summed according to when they occurred in the tracking period either immediately post-release (“post-release”) or after individuals had left the tracking region and returned (“returning”). Sample size for (**a**,**b**) is 2 tags and for (**c**–**e**) 25 tags. Map created using R^37^ and RStudio^38^.

Of the 25 ABT tracked for 300 days or more, 22 fish (88%) were tracked returning to the Channel between 32 and 369 days after release (mean = 245 ± 114 days), with most (*n* = 14, 58%) individuals returning for the first time in July and August the year after tagging (Fig. 3). Individuals that returned before July remained for short periods (3–39 days; mean = 17 ± 17 days), before leaving the region again. No individuals were recorded in the Channel between April and June in any tracking year. Three individuals did not conduct return migrations to the Channel or Celtic Seas regions in the year after tag attachment and ended their deployments northwest of Ireland (*n* = 3; 12%). Two individuals tracked for more than 365 days, provided continuous estimates of residency in the Channel in the second tagging year. The first individual (Fig. S4) was tracked for 481 days, returning to the Channel on the 23rd of July 2021 before leaving on the 4th of December 2021 (135 days in the Channel). A second individual (Fig. S5) was tracked for 708 days and returned to the Channel on the 15th of July and stayed either in the Channel or Celtic Seas region until the 6th of December 2021 (145 days in the Channel).

During the off-shelf phase, ABT were most likely to be in either the French EEZ in the BoB (grand mean probability of occurrence 0.27 ± 0.19) or the High Seas (0.34 ± 0.21). The highest probability density during this phase occurred in the eastern BoB, a core area in the complete migratory cycle (Fig. 2).

### Migrations during the spawning season

In total, 25 fish were tracked over at least one full spawning season, with three more ending their deployments off the Balearic Islands after 283 days, and between the Straits of Gibraltar and the northwestern European Shelf after 292, and 297 days. Sixteen ABT (57% of 28) were tracked entering the Med., crossing the Strait of Gibraltar between the 5th of May and the 14th of June (mean = 23rd of May) and remaining for 31 to 77 days (mean = 50 days) before 15 were tracked exiting between the 1st and the 26th of July (mean = 11th July). Twelve ABT (43%) remained in the BoB between May and July and did not visit a known spawning ground. These individuals were significantly smaller at release (mean = 174 ± 21 cm CFL) than those that entered the Med. (213 ± 19 cm CFL; Wilcoxon rank sum test, *r* = 0.64, W = 171, = <0.001). Growth equations indicate the mean size of entry to the Med. was 220 ± 19 cm CFL (minimum 184 cm CFL) but not all ABT larger than 184 cm CFL on the 23rd of May (mean date of entry) entered the Med., and 27% (*n* = 6) remained in the Atlantic Ocean during the spawning season. A 241 cm CFL (at release) individual tracked for 708 days (Fig. S5) remained in the Atlantic Ocean during the spawning season in the first tracking year but entered the Med. in the subsequent tracking year (estimated size at entry = 253 cm CFL). When in the Med., individuals visited known spawning grounds off the Balearic Islands (Spain; grand mean probability of occurrence = 0.19 ± 0.19), and Tunisian (0.12 ± 0.03), Libyan (0.16 ± 0.11) and Italian coasts (0.13 ± 0.11). Fish that remained in the Atlantic Ocean in the months of May to July (*n* = 12) were most likely to be in the French (grand mean probability of occurrence = 0.21 ± 0.2) or Spanish EEZs in the BoB (0.22 ± 0.19) or the High Seas (0.21 ± 0.22). The highest location densities for these fish occurred in the southeastern bight of the BoB, particularly in July. Fish leaving the Med. took 20 ± 12 days to return to waters of the Channel and Celtic Seas (range = 11 to 56 days) at a mean speed of 0.5 ± 0.18 body lengths per second.

### Vertical habitat use

**Fig. 4:**
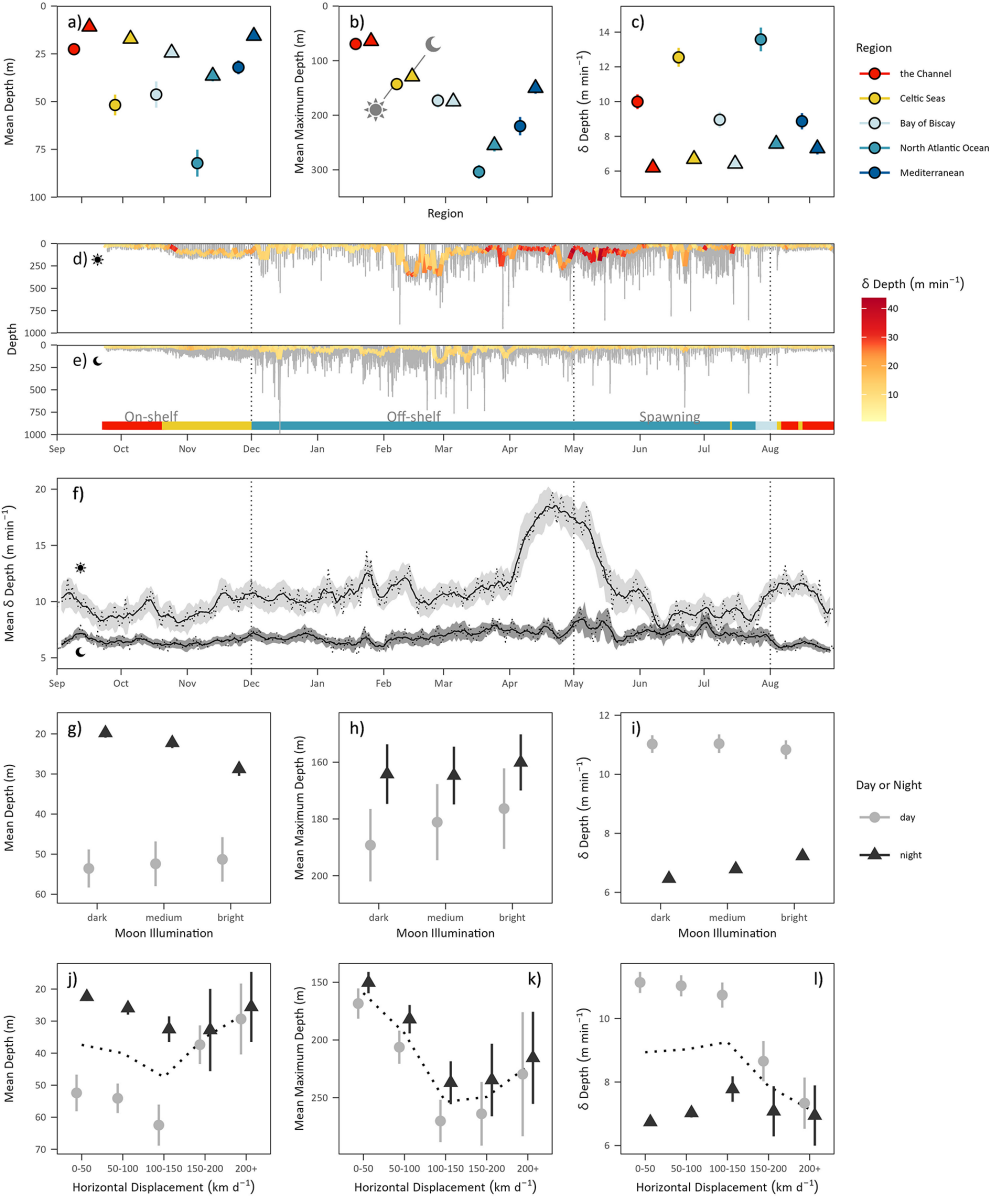
Diving behaviour of 19 tracked Atlantic bluefin tuna from recovered PSAT archives. (**a**–**c**) Box and whisker plots showing geographic and day/nighttime variation in grand means of mean depth occupied (m), maximum depth reached (m) and vertical movement rate. (**d**,**e**) Representative day (**d**) and nighttime (**e**) diving behaviour of 20P0089 (229 cm CFL at release) over 348 days. Time series shows raw diving behaviour as a grey line, with hourly mean depth overplotted and coloured by vertical movement rate. The inset horizontal bar denotes geographic region occupied, and annotations and vertical dotted lines denote tracking phase. (**f**) Time series of mean vertical movement rate for day and nighttime periods for tracked ABT pooled. Raw data are plotted as a dotted line with weekly smoothed data overplotted. Grey polygons denote ± 1 Standard Error. (**g**–**i**) Point plots showing day/nighttime variation in grand means of mean depth occupied (m), maximum depth reached (m) and vertical movement rate by moon illumination category. (**j**–**l**) Box and whisker plots showing day/nighttime variation in grand means of mean depth occupied (m), maximum depth reached (m) and vertical movement rate by horizontal displacement category. Dotted line denotes the mean. Where shown, error bars denote ± 1 Standard Error^36–38^.

High resolution dive data recovered from 19 tags (*n* = 6,043 days) revealed ABT occupied waters between the surface and 1,611 m depth (Fig. S7) but spent most time in the top 20 m of the water column in both daytime (49 ± 6% of time) and nighttime periods (71 ± 6% of time), in all geographic regions and time periods (Fig. S8). Total daily vertical movements varied between 511 and 39,428 m day^− 1^ (mean = 12,663 ± 4935 m.day^− 1^). Mean depth, maximum depth and VMR varied depending on the daytime/nighttime period, level of lunar illumination, horizontal displacement, tracking phase and region occupied by the ABT (Fig. 4; Table S6).

Overall, ABT mean depth (daytime = 48 ± 26 m vs. nighttime 20 ± 10 m), maximum depth (daytime = 180 ± 82 m vs. nighttime = 156 ± 67 m) and VMR was significantly greater during the daytime than at nighttime (daytime = 11.2 ± 2.1 m min^− 1^ vs. nighttime = 6.8 ± 0.7: GLMMs, P ≤ 0.001; Fig. 4a–f). Furthermore, ABT occupied significantly deeper depths on average and conduced more vertical movements when the moon was brightest (Fig. 4g and i; see Fig. S10 for Tukey comparisons), but there was no effect of lunar illumination on maximum depth (Fig. 4h; GLMM: Χ^2^_2_ = 0.2, *P* = 0.87). As ABT conducted faster horizontal movements they occupied significantly shallower mean depths (GLMM: Χ^2^_1_ = 16.2, P = < 0.001) and reduced VMRs (Χ^2^_1_ = 22.1, P = < 0.001), with the largest differences occurring during daytime periods (Fig. 4j–l). Maximum depth was not significantly affected by horizontal movement speed (Χ^2^_1_ = 0.4, *P* = 0.6).

We found a significant interaction between geographic sea region inhabited and tracking phase on mean depth (GLMM: Χ^2^_12_ = 289.8, P = < 0.001), maximum depth (Χ^2^_12_ = 850.9, P = < 0.001) and VMR (Fig. S11; Χ^2^_12_ = 93, P = < 0.001). Mean depths occupied and maximum depths were consistently the deepest in the North Atlantic Ocean region (grand mean depth = 59 ± 27 m; grand mean maximum depth = 294 ± 28 m; Table S7), and during the off-shelf phase for all regions (grand mean depth = 43 ± 24 m; grand mean maximum depth = 176 ± 87 m). Following this, the shallowest mean depths occupied, and maximum depths were observed in the Channel (grand mean depth = 18 ± 7 m; grand mean maximum depth = 74 ± 8 m) and Celtic Seas regions (grand mean depth = 26 ± 19 m; grand mean maximum depth = 113 ± 20 m). Rates of vertical movement were highest during the off-shelf (daytime = 13.6 ± 3.9, nighttime = 7.4 ± 1.4 m min^− 1^) and spawning phase in the North Atlantic Ocean (daytime = 14.6 ± 3, nighttime = 8.9 ± 2.4 m min^− 1^) and the lowest in the BoB and the Med., particularly during the spawning season (BoB: daytime = 7.5 ± 2.8 m min^− 1^; nighttime = 6.6 ± 0.5 m min^− 1^; Med.: daytime = 8.9 ± 1.3 m min^− 1^; nighttime = 7.3 ± 1 m min^− 1^). Continuous time series of VMR data (for all years pooled; Fig. 4f) revealed that the highest mean VMR occurred during the off-shelf and on-shelf phases during April and May in all study years. This behaviour occurred predominantly on the continental shelf edge between the North Atlantic Ocean and Celtic Seas regions (Fig. S12). Here, ABT made daytime oscillatory dives (daytime mean VMR = 22 ± 4 m min^1^; nighttime mean VMR = 9 ± 4 m min^− 1^) at a mean depth of 102 ± 90 m (nighttime depth, 21 ± 40 m).

## Discussion

Here we report on the first ABT tracked from waters of the Channel, a re-established seasonal hotspot between August and December. Tracking data reveal inter-annual fidelity in all study years, including a tag attached for 2-years detailing multi-annual fidelity. We show how movements connect aggregations within the Channel and the Celtic Seas with numerous, temporally stable, seasonal hotspots such as the BoB, the Central North Atlantic and numerous spawning grounds in the Mediterranean Sea. Importantly, we show (1) limited connectivity with Nordic regions, which ABT have also re-occupied in recent years^9,50^; and (2) ABT tracked from waters off the UK predominantly occupy eastern-stock management regions, consistent with other studies on larger ABT^28,35,50^.

Archival data shed light on how ABT use the ocean vertically, and how this varies geographically and temporally. Generally, data suggest that both lunar and solar illumination influence vertical habitat use, with individuals occupying greater mean depths, diving to greater maximum depths, and conducting more vertical movements during the daytime and when lunar illumination was greatest. This relationship is, however, not evident during periods of fast dispersal, and as horizontal movement rates increased, individuals occupied shallower depths on average and reduced vertical movements during the daytime.

### How important is the channel to ABT?

The Channel is a warm (15–20 °C during summer and autumn^51^), productive (up to 8 mg Chl-a m^− 3^^52^), boundary region separating oceanic and neritic waters. It is inhabited by both boreal and warm temperate prey species^53^, and large predators of these prey, such as blue (*Prionace glauca*) and porbeagle sharks (*Lamna nasus*^54,55^). Only one ABT had previously been tracked into the Channel^21^ with all other individuals tracked in other studies avoiding the region^1,18–20,56^. Our tagging data show that ABT resided in, and returned to, waters of the Channel in all tracking years, with records indicating that these aggregations have been present since at least 2014^7^. Two ABT, tracked for 481 and 708 days, provide an unbiased (either by behavioural alteration due to tagging or tag pop-up dates) corroboration of seasonal residency and fidelity of ABT, with both individuals returning to the tagging site in July the year after tagging and leaving again in December. Drivers for this could be the proliferation of warm water species in response to the subtropicalization of the northwest European shelf^57^, including European sardine (*Sarda pilchardus*) and European anchovy (*Engraulis encrasicolus*), both of which are favoured prey of ABT^58^ and may attract them to the region. Additionally, niche modelling has highlighted waters of the Channel as suitable abiotic habitat for small (5–25 kg) and large (> 25 kg) ABT since the 1990s^13,59^. Finally, since the late 2000s there has been a strong increase in the size of the eastern ABT stock^5^, which may have resulted in ABT returning to waters off the UK and CI. Whilst the drivers of the re-occurrence may be complex, our data confirm that waters of the Channel now host multiple size cohorts of ABT during the summer and autumn months, and that these individuals return after completing extensive annual migrations. Future research should aim to provide clearer linkages between ABT and the biotic and abiotic factors that attract them to the study region, with a view to better understanding the regional ecosystem that they now, once again, play a key role in as apex predators.

### Open ocean movements of ABT

When not resident in the Celtic Seas and Channel regions, ABT dispersed widely in the North Atlantic during the rest of the year. Much of this time was spent in the BoB, the Central North Atlantic, and known spawning grounds in the western and central Mediterranean Sea. Several studies have been conducted on smaller ABT in the BoB^19,60,61^, where they are known to occur between June and October. However, most tracking data to date has suggested that larger ABT are generally absent from the BoB during this time, despite some fisheries operating in the BoB catching larger individuals in the region between July and August^60^. Here we show ABT migrate northwards from the BoB to the Channel and Celtic Seas, or to areas off northwest Ireland during these months with peak occupancy in the BoB between December and July (outside the periods when fisheries are active). Consequently, larger ABT (158–242 cm CFL) are largely absent from the BoB during the peak sampling season. This (1) may explain their absence from other scientific datasets, and (2) highlights the value of tracking ABT from aggregations throughout their range, with our data showing that the BoB is an important winter and spring habitat for larger ABT.

A key topic for ICCAT concerning the spatial fisheries management strategy for ABT is how the spatial ecology of ABT changes as individuals grow. Research has shown both that fish smaller than 200 cm CFL tracked from Malta to not leave the Mediterranean^21^, whereas smaller bodied ABT (64 to 110 cm FL) tracked from the BoB conducted trans-Atlantic migrations, traversing eastern and western management regions^19^. Though the fish tracked in the present study were larger (minimum in the present study 153 cm CFL), none conducted trans-Atlantic migrations, and only four (of 45 fish; 9%) crossed the 45° W meridian (similar to ABT tagged in Ireland and Denmark;^28,35,50^). This has also been documented in the western Atlantic, with 49 large (243–302 cm CFL) ABT tracked from the Gulf of St. Lawrence, Canada, all remaining within western stock management regions for the entire annual migratory cycle^23^. This may indicate that the adult portion of the eastern stock already present on eastern Atlantic foraging grounds conduct fewer longitudinal movements than smaller bodied conspecifics. This will of course need further investigation using electronic tagging at sites in the northeast Atlantic.

The Central North Atlantic is well documented as a hotspot for marine predators, with high productivity driven by mesoscale oceanographic features attracting sharks^62–64^, sea birds from both North and Southern hemisphere populations^65^ and ABT tracked from the BoB^19^, Ireland^28,35^, the Kattegat^50^, the Gulf of St. Lawrence^23^, the Outer Banks^1,18^ and now the Channel. Despite mixing of ABT from multiple different coastal foraging aggregations in the Central North Atlantic, we observed limited connectivity between the ABT tagged in the Channel and foraging areas off Ireland^28,35^ and no connectivity to foraging areas in the North Sea^9,50^. Larger ABT can tolerate cooler temperatures and, therefore, more northerly regions^1,66^. This is evident in the increase in median ABT size with the latitude of foraging aggregations, from 197 cm CFL in the present study, to 205 cm off Ireland^28^, 241 cm off Sweden and Denmark^67^ and 255 cm off Norway^68^. It could be that fish in the present study may eventually shift to more northerly summer-autumn habitats as they grow larger. Alternatively, it could be that the eastern ABT stock exists as numerous spatially discrete units, rather than their distribution being structured by age or size. The tracking durations in this study (2-years, the maximum operational duration of MiniPAT tags;^69^), limited our ability to detect ontogenetic distributional shifts or the presence of spatially discrete sub units, but future tracking with longer duration acoustic or implantable archival tags (both up to 5 years duration, e.g.^1^) could reveal longer term shifts in habitat use.

### Spawning migrations

In our study, ABT only moved into the Mediterranean Sea during the spawning season, suggesting that these individuals originate there and thus belong to the eastern ABT stock^4,70^. Furthermore, preliminary genetic assignment of ABT tagged in this study (2018 and 2019) strongly suggest they are of eastern stock origin (McKeown, unpubl. data). The mean size of entry to the Mediterranean Sea in this study was 220 ± 19 cm CFL (minimum estimated 184 cm CFL), with six smaller ABT (range 158–166 cm CFL) remaining in the BoB during the spawning season yet exhibiting similar spatial habits to spawning individuals for the remainder of the year. Although ICCAT considers that 50% of the population matures by 115 cm CFL, the size for 100% maturity in eastern ABT is 135 to 158 cm fork length (~ 5 years old^41,71,72^). Our data concurs with other studies that indicate ABT in the Atlantic do not migrate into the Mediterranean Sea until they are larger than this (≥ 200 cm CFL in^1^; ≥ 206 cm CFL in^35^). This is further corroborated by the size of adult ABT captured in the trap fisheries off Portugal and Morocco, targeting ABT on their spawning migrations into the Mediterranean Sea, with catches comprised predominantly of ABT > 150 cm CFL^73,74^. This may reflect a difference in rate of maturation for ABT that leave the Mediterranean Sea as juveniles, or even alternate spawning grounds (as has been shown in the western Atlantic^75^). This latter hypothesis could also explain why a 241 cm individual tracked for 708 days did not enter the Mediterranean Sea in the first spawning season but did in the second. However, as we only tracked one fish over multiple spawning seasons this requires further research. Nonetheless, potential differences in the age at maturation or alternative spawning ground usage could have consequences for stock assessment models (i.e^41,71,76^), and in turn have an influence on estimates of spawning stock biomass. How much influence would however depend on the number of ABT now migrating to the Channel which is currently unknown.

### Vertical habitat use

The ocean environment is three dimensional, and pelagic predators conduct extensive vertical movements away from surface waters for foraging^77^, navigation^78^ and thermoregulation^79^. Our results show that lunar and solar illumination affect the vertical habitat use of ABT (see also^80,81^). Here, tracked ABT consistently increased the depth they occupied and their rates of vertical movement during the daytime, and on nights when lunar illumination was brightest. This likely indicates foraging at depth during the daytime and full moon when light penetrates deeper, allowing exploration of the significant resources at depth^76^. This has also been shown for yellowfin tuna (*Thunnus albacares*^82^) with deeper depths occupied during the daytime. However, during fast dispersal this general relationship breaks down, with ABT occupying shallower depths on average and reducing VMRs during the daytime. Southern bluefin tuna (*Thunnus maccoyii*) make “spike” dives (sharp angled descents and ascents) that may represent attempts to gain navigational cues by sampling the water column^83^. Here we show that maximum depths did not increase during periods of fast dispersal when we might expect navigational behaviours to increase. This may indicate either that navigational dives are conducted continually, and are thus not affected by movement mode, or that these dives serve an alternate purpose to navigation.

Periods where behaviour differs from the “normal” can aid in identifying key life history events. Here, we identify high mean VMRs west of France in the North Atlantic Ocean in April and May and particularly low mean VMRs coupled with a shallowing of mean depth in both the BoB and in the Mediterranean between May and July (the spawning season). Although challenging to confirm, the timing of these behaviours indicate that they could relate to reproduction and may have implications for population productivity. Initially, most of the highest VMRs identified occurred between April and May in a region west of France (Fig. S11), which may signify ABT foraging on a predictable (i.e. spatio temporally stable) food resource. Mackerel are an important prey species of ABT^84^. In other regions, such as the Gulf of St. Lawrence, ABT have been shown to time their migrations to take advantage of prey as they aggregate to spawn^23^. Western spawning Northeast Atlantic mackerel (*Scomber scomber*) aggregate to reproduce in a similar region and time of year to where we document the highest rates of vertical movement of ABT^85^. Consequently, it could be that ABT target mackerel as they aggregate, providing a predictable feeding opportunity prior to spawning. Whilst this predator-prey relationship is challenging to substantiate, this behaviour occurring so close to the reproductive season warrants deeper investigation to understand its function and significance to mature ABT.

Second, we observed ABT reduce their mean depth and VMRs during the spawning season in the BoB with similar patterns in the Mediterranean Sea. Spawning ABT conduct high VMR behaviours intersecting the thermocline between midnight and sunrise^20,29,35,50^. Our results instead indicate a more general behavioural alteration during the spawning period, with an overall reduced VMR during the spawning season, and perhaps increased only during periods when oocytes are released into the water column (e.g^20^). Although beyond the scope of this study, a more detailed analysis of spawning behaviour would provide valuable insights into this key life history stage, which has important implications for fisheries management and conservation.

## Conclusion

The conservation of ABT is dependent on appropriate management of the Atlantic wide commercial fishery by ICCAT^2^, which knowledge on their complex spatial ecology contributes to. The recent increase of ABT in the northeast Atlantic highlights this complexity, as ABT are now once again found in regions from which they have been absent for many decades. The results presented here provide a first perspective on the spatial ecology of ABT that are found off the UK, and how this compares with what is already known about eastern ABT. Our data indicate that waters of the Channel are occupied by ABT for much of the year, with peak occurrences between August and November. Individuals tracked from the Channel are faithful to spatially discrete eastern-stock management regions, occupying waters east of 45°W and visit numerous known Mediterranean Sea spawning grounds, with little to no connectivity to western-stock regions. Within eastern stock regions, ABT were most likely to be found either in the Channel, or the south-eastern bight of the BoB, indicating a key link between re-occupied regions and temporally stable aggregation sites in the BoB.

Our data and analysis reveal that the spatial ecology of eastern ABT may be more complex than previously assumed, with potential knock-on effects for how stock assessment models are structured, and therefore on their outputs. The research detailed here indicates that ABT now present once again in the Channel are comprised of individuals that may offer valuable insight into the dynamics of the eastern spawning stock. Consequently, future efforts should aim to shed light on the mechanisms that underpin the complex distribution of this emblematic predator, with a focus on key life history traits such as spawning behaviour, migratory philopatry and ontogenetic change in distribution.

## Electronic supplementary material

Below is the link to the electronic supplementary material.

Supplementary Material 1

## Data Availability

Datasets generated during the current study are available from the authors on reasonable request, by contacting the corresponding author.
