# Publisher Correction: Annual migrations, vertical habitat use and fidelity of Atlantic bluefin tuna tracked from waters off the United Kingdom

**DOI:** 10.1038/s41598-025-90365-w

**Published:** 2025-02-19

**Authors:** Thomas W. Horton, Francis C. T. Binney, Samantha Birch, Barbara A. Block, Owen M. Exeter, Francesco Garzon, Alex Plaster, David Righton, Jeroen van der Kooij, Matthew J. Witt, Lucy A. Hawkes

**Affiliations:** 1 Environment and Sustainability Institute, University of Exeter, Penryn, TR10 9FE UK; 2 Hatherly Laboratories, University of Exeter, Prince of Wales Road, Exeter, EX4 4PS UK; 3 Centre for Ecology and Conservation, University of Exeter, Penryn Campus, Penryn, Cornwall, TR10 9FE UK; 4 Government of Jersey Marine Resources, Natural Environment, Howard Davis Farm, Trinity, Jersey; 5 Centre for Environment, Fisheries and Aquaculture Science, Pakefield Road, Lowestoft, NR33 0HT UK; 6 Department of Oceans, Stanford University, Hopkins Marine Station, Pacific Grove, CA USA; 7 School of Environmental Sciences, University of East Anglia, Norwich, NR4 7TJ UK

Correction to: *Scientific Reports* 10.1038/s41598-024-80861-w, published online 02 January 2025

In the original version of this Article, Thomas W. Horton was omitted as a corresponding author. Correspondence and requests for materials should also be addressed to: t.horton@exeter.ac.uk.

The original Article has been corrected.

