# Supplementary material for: Annual migrations, vertical habitat use and fidelity of Atlantic bluefin tuna tracked from waters off the United Kingdom
Source: Sci Rep. 2025 Jan 2;15:293. doi: 10.1038/s41598-024-80861-w (PMC11696515; doi:10.1038/s41598-024-80861-w)
Supplement: Supplementary file 1 — Supplementary Material 1 [file 41598_2024_80861_MOESM1_ESM.docx]

**Supplement**

**Annual migrations, vertical habitat use and fidelity of Atlantic bluefin tuna tracked from waters off the United Kingdom**

Thomas W. Horton^1-3^, Francis C. T. Binney^4^, Samantha Birch^5^, Barbara A. Block^6^, Owen M. Exeter^3^,
Francesco Garzon^1^, Alex Plaster^4^, David Righton^5,7^, Jeroen van der Kooij^5^, Matthew J. Witt^1,2^ and
Lucy A. Hawkes^1^

^1^ Environment and Sustainability Institute, University of Exeter, Penryn TR10 9FE

^2^ Hatherly Laboratories, University of Exeter, Prince of Wales Road, Exeter, EX4 4PS, UK

^3^ Centre for Ecology and Conservation, University of Exeter, Penryn Campus, Penryn, Cornwall, TR10 9FE, UK

^4^ Government of Jersey Marine Resources, Natural Environment, Howard Davis Farm, Trinity, Jersey

^5^ Centre for Environment, Fisheries and Aquaculture Science, Pakefield Road, Lowestoft NR33 0HT, UK

^6^ Department of Oceans, Stanford University, Hopkins Marine Station, Pacific Grove, California, USA

^7^ School of Environmental Sciences, University of East Anglia, Norwich NR4 7TJ, UK

**Table S1. Metadata for Wildlife Computers MiniPATs attached to Atlantic bluefin tuna and included in spatial analyses.** For “Entry to Med.”, “Atl.” denotes a fish that remained in the open Atlantic Ocean during the putative spawning season and “Prem.” denotes a tag that detached prior to the spawning season (and thus affinity to the Mediterranean Sea could not be assessed). “^Δ^” denotes a tag that was not attached for 300 days (i.e. a premature detachment) but that was tracked into the Mediterranean Sea. “ε” denotes individuals that were preliminarily assigned to the eastern Atlantic ABT stock using mtDNA. “*” denotes a tag that was physically recovered. “^” denotes a dataset that was used in analyses investigating the influence of movement speed on GPE3 track reconstruction as detailed in Horton *et al.* [35] and Figure S1.

|  | Deployment | | | |  | Pop-up | | | |  |  |  |
| --- | --- | --- | --- | --- | --- | --- | --- | --- | --- | --- | --- | --- |
| Tag | **Date** | **Lat** | **Lon** | **Curved Fork Length (cm; Age)** |  | **Date** | **Lat** | **Lon** | **Days at Large**  **(d; % data)** | **Entry to**  **Med.** | **Exit from**  **Med.** | **Days in Med.** |
| 16P1483^*^ε^ | 25/10/2018 | 50.12 | -4.79 | 206 (8) |  | 14/09/2019 | 50.14 | -3.47 | 324 (100) | 19-May | 05-Jul | 47 |
| 18P0808^*^ε^ | 23/08/2019 | 49.9 | -5.04 | 214 (8) |  | 22/08/2020 | 50.04 | -4.4 | 366 (100) | 08-Jun | 14-Jul | 36 |
| 18P0837^*^ε^ | 02/09/2019 | 49.92 | -5.06 | 181 (6) |  | 02/09/2020 | 55.4 | -9.71 | 366 (100) | 01-Jun | 14-Jul | 43 |
| 17P1004^*^ε^ | 03/09/2019 | 50.04 | -4.99 | 203 (8) |  | 02/09/2020 | 48.9 | -4.58 | 366 (100) | 05-May | 06-Jul | 62 |
| 19P0206^*^ε^ | 02/10/2019 | 50.14 | -4.71 | 221 (9) |  | 28/09/2020 | 50.15 | -4.77 | 362 (100) | 08-May | 24-Jul | 77 |
| 19P0175^*^ε^ | 05/10/2019 | 50.09 | -4.96 | 218 (9) |  | 28/09/2020 | 50.17 | -4.71 | 359 (100) | 26-May | 11-Jul | 46 |
| 18P0938*^ε^ | 22/10/2019 | 51.37 | -6.18 | 237 (10) |  | 21/09/2020 | 55.28 | -9.21 | 334 (100) | 06-Jun | 07-Jul | 31 |
| 18P0932^*^ε^ | 23/10/2019 | 51.37 | -6.18 | 238 (10) |  | 21/09/2020 | 51.31 | -6.49 | 334 (100) | 15-May | 13-Jul | 59 |
| 20P0085 | 08/09/2020 | 50.11 | -4.85 | 214 (8) |  | 07/09/2021 | 57.06 | -7.91 | 364 (31) | 28-May | 15-Jul | 48 |
| 20P0084* | 09/09/2020 | 50.11 | -4.95 | 229 (9) |  | 07/09/2021 | 50.1 | -4.55 | 362 (100) | 11-May | 14-Jul | 64 |
| 20P1136* | 12/09/2020 | 50.1 | -4.9 | 194 (7) |  | 15/09/2021 | 49.16 | -3.44 | 367 (100) | 19-May | 12-Jul | 54 |
| 20P1137* | 16/09/2020 | 50.11 | -4.83 | 201 (7) |  | 15/09/2021 | 49.46 | -3.17 | 364 (100) | 17-May | 04-Jul | 48 |
| 20P0056* | 27/09/2020 | 50.15 | -4.91 | 241 (10) |  | 05/09/2022 | 50.34 | -4.5 | 708 (100) | 20-May | 01-Jul | 42 |
| 20P1178 | 14/09/2021 | 50.03 | -4.64 | 207 (8) |  | 08/07/2022 | 40.71 | -10.12 | 297 (74)^Δ^ | 17-May | 03-Jul | 47 |
| 18P0812^*^ε^ | 02/11/2018 | 50.16 | -4.82 | 175 (6) |  | 21/08/2019 | 48.21 | -6.54 | 292 (100) ^Δ^ | 14-Jun | 26-Jul | 42 |
| 20P1139 | 16/09/2020 | 50.13 | -4.85 | 222 (9) |  | 26/06/2021 | 38.06 | 0.39 | 283 (49) ^Δ^ | 05-Jun | - | - |
| 16P1553 ^ε^ | 09/10/2018 | 50.13 | -4.67 | 191 (7) |  | 24/08/2019 | 49.26 | -2.86 | 319 (59) | Atl. | - | - |
| 17P0786^*^ε^ | 22/10/2018 | 50.12 | -4.81 | 158 (5) |  | 14/09/2019 | 48.34 | -6.29 | 327 (100) | Atl. | - | - |
| 16P1898^ε^ | 23/10/2018 | 50.1 | -4.86 | 163 (5) |  | 14/09/2019 | 49.32 | -2.95 | 326 (46) | Atl. | - | - |
| 18P0835^ε^ | 25/08/2019 | 49.93 | -5.04 | 161 (5) |  | 24/08/2020 | 49.95 | -8.01 | 365 (27) | Atl. | - | - |
| 19P0216^*^ε^ | 23/10/2019 | 50.13 | -4.81 | 198 (7) |  | 28/09/2020 | 50.15 | -4.29 | 341 (100) | Atl. | - | - |
| 19P0137^*^ε^ | 18/11/2019 | 50.15 | -4.82 | 166 (5) |  | 28/09/2020 | 50.08 | -4.13 | 315 (100) | Atl. | - | - |
| 20P0094* | 21/09/2020 | 50.13 | -4.89 | 190 (7) |  | 15/01/2022 | 43.68 | -2.2 | 481 (100) | Atl. | - | - |
| 20P0089* | 23/09/2020 | 50.22 | -4.74 | 220 (9) |  | 07/09/2021 | 50.13 | -4.52 | 348 (100) | Atl. | - | - |
| 21P0467* | 05/09/2021 | 49.07 | -2.4 | 179 (6) |  | 11/09/2022 | 49.27 | -3.3 | 371 (100) | Atl. | - | - |
| 21P0341* | 07/09/2021 | 49.05 | -2.33 | 154 (5) |  | 11/09/2022 | 48.99 | -2.77 | 368 (100) | Atl. | - | - |
| 21P0399* | 12/10/2021 | 50.08 | -4.84 | 201 (7) |  | 13/10/2022 | 50.16 | -2.96 | 366 (100) | Atl. | - | - |
| 21P0397 | 12/10/2021 | 50.05 | -4.8 | 168 (5) |  | 13/10/2022 | 48.89 | -4.37 | 366 (36) | Atl. | - | - |
| 17P0711^ε^ | 04/10/2018 | 50.01 | -5.07 | 193 (7) |  | 11/02/2019 | 39.51 | -40.01 | 130 (81) | Prem. | - | - |
| 16P2192* | 08/10/2018 | 50.12 | -4.75 | 184 (6) |  | 22/01/2019 | 43.76 | -3.59 | 106 (100) | Prem. | - | - |
| 17P0960^ε^ | 08/10/2018 | 50.13 | -4.88 | 166 (5) |  | 09/04/2019 | 40.56 | -41.15 | 183 (50) | Prem. | - | - |
| 18P0838^ε^ | 13/08/2019 | 49.95 | -5.16 | 230 (9) |  | 09/12/2019 | 47.87 | -5.57 | 118 (40) | Prem. | - | - |
| 16P1231*^ε^ | 23/08/2019 | 49.89 | -5.03 | 212 (8) |  | 15/05/2020 | 49.88 | -15.95 | 266 (100) | Prem. | - | - |
| 18P0836^ε^ | 23/08/2019 | 49.89 | -5.02 | 184 (6) |  | 23/02/2020 | 38.53 | -14.91 | 184 (28) | Prem. | - | - |
| 16P2365*^ε^ | 25/08/2019 | 49.9 | -5.04 | 199 (7) |  | 25/12/2019 | 44.81 | -1.57 | 122 (100) | Prem. | - | - |
| 19P0136^ε^ | 13/10/2019 | 50.09 | -5.02 | 215 (8) |  | 19/03/2020 | 42.78 | -21.16 | 158 (51) | Prem. | - | - |
| 20P0086 | 09/09/2020 | 50.1 | -4.88 | 204 (8) |  | 19/05/2021 | 48 | -11.21 | 252 (43) | Prem. | - | - |
| 20P0060* | 09/09/2020 | 50.1 | -4.94 | 197 (7) |  | 08/01/2021 | 47.5 | -40.31 | 121 (100) | Prem. | - | - |
| 20P0083 | 16/09/2020 | 50.08 | -4.86 | 185 (6) |  | 05/12/2020 | 44.95 | -3.04 | 80 (51) | Prem. | - | - |
| 20P0055 | 22/09/2020 | 50.1 | -4.94 | 216 (8) |  | 31/12/2020 | 45.68 | -2.41 | 99 (51) | Prem. | - | - |
| 20P1175 | 23/09/2020 | 50.24 | -4.71 | 242 (10) |  | 23/05/2021 | 45.79 | -5.26 | 241 (44) | Prem. | - | - |
| 21P0469 | 04/09/2021 | 49.1 | -2.44 | 154 (5) |  | 03/02/2022 | 44.7 | -2.21 | 152 (66) | Prem. | - | - |
| 21P0468* | 04/09/2021 | 49.08 | -2.34 | 153 (5) |  | 23/12/2021 | 50.08 | -0.57 | 110 (100) | Prem. | - | - |
| 20P0856 | 14/10/2021 | 49.99 | -4.76 | 241 (10) |  | 20/12/2021 | 22.2 | -17.38 | 67 (87) | Prem. | - | - |
| 20P0095 | 14/10/2021 | 50.08 | -4.66 | 175 (6) |  | 12/04/2022 | 36.69 | -56.09 | 180 (54) | Prem. | - | - |
| Mean ± 1 S.D.  Range | | | | 198 ± 26 cm |  |  |  |  | 282 ± 124 d | 23 May ± 12 d | 11 Jul ± 7 d | 50 ± 12 d |
|  |  |  |  | 153-242 cm |  |  |  |  | 67-708 d | 5 May - 14 Jun | 1 Jul - 26 Jul | 31-77 d |

**
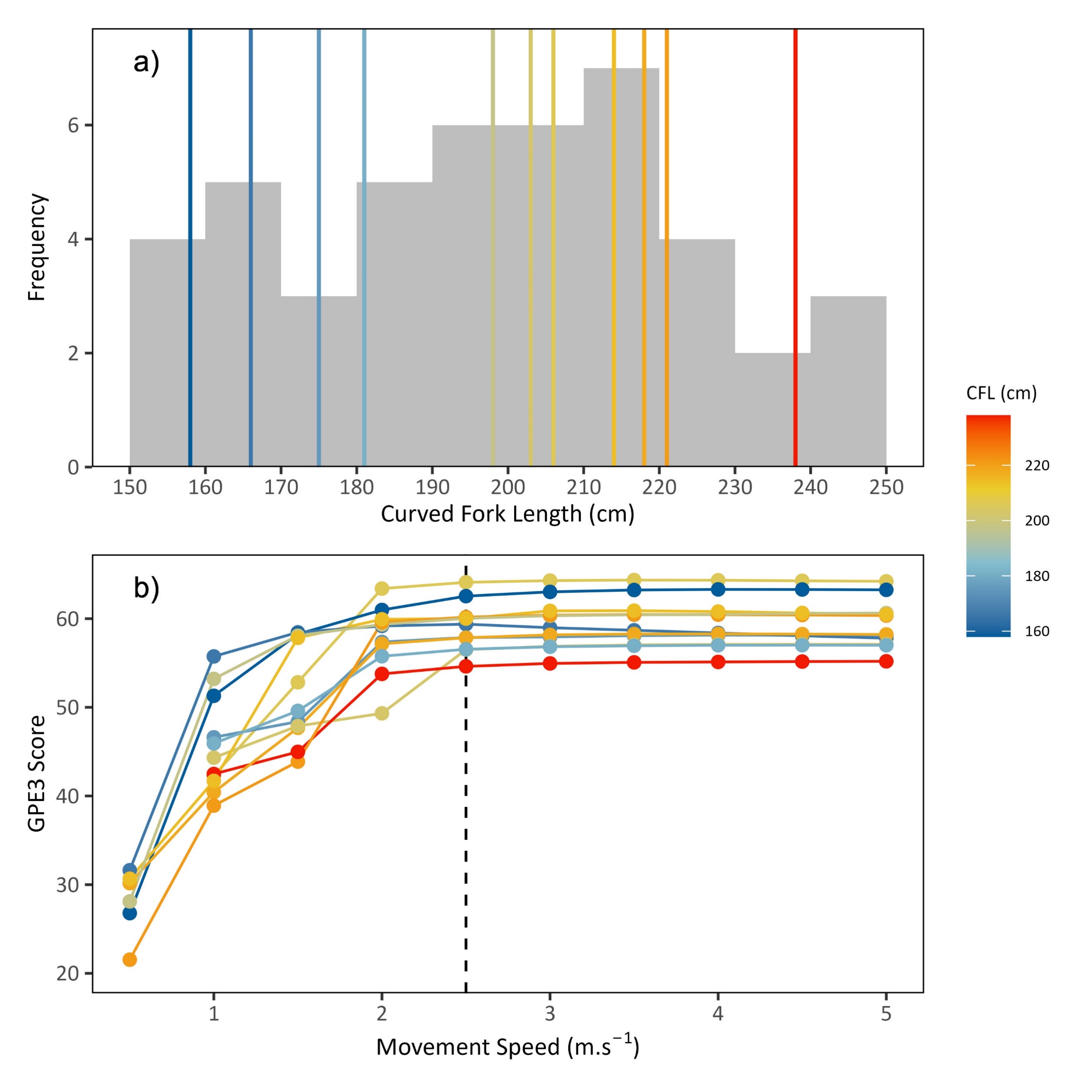
**

**Figure S1. Comparison of GPE3 scores for different model movement speeds for a range of different sized ABT tracked in this study.** a) Length-frequency histogram for the ABT tracked in this study. Overlaid vertical lines denote individual ABT for which model movement speed analysis was conducted coloured by curved fork length. b) Line plot with one line per individual identified on “a” with the same colours used. Each individual data point denotes an individual GPE3 run. Vertical dashed line denotes the 2.5 m s^-1^ movement speed adopted in this study.


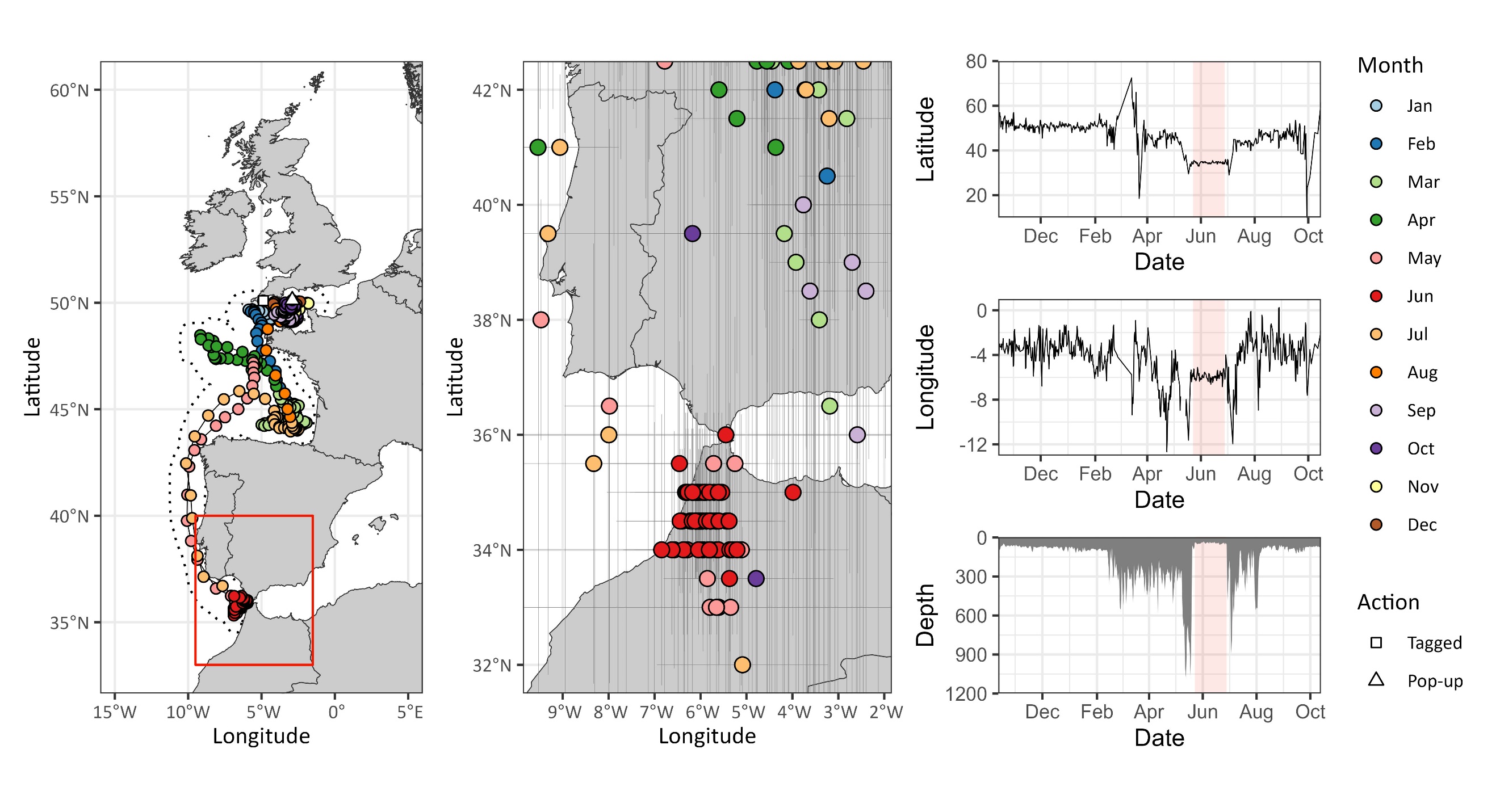


a)

b)

c)

d)

e)

**Figure S2. Reconstructed movements and diving behaviour for 21P0399 supporting hypothesis for capture in trap.** a) Map showing GPE3 output of all locations from the deployment with the area where individual was considered to be in a trap as a red box. b) Map zoomed in on the trap region showing output from light based geolocation model only (i.e. GPE2) with error bars. c-d) Time series of latitude and longitude showing the trap period as a red polygon. e) Depth plot showing maximum depth over time indicating restricted vertical movements during the eastern spawning period.

.

**
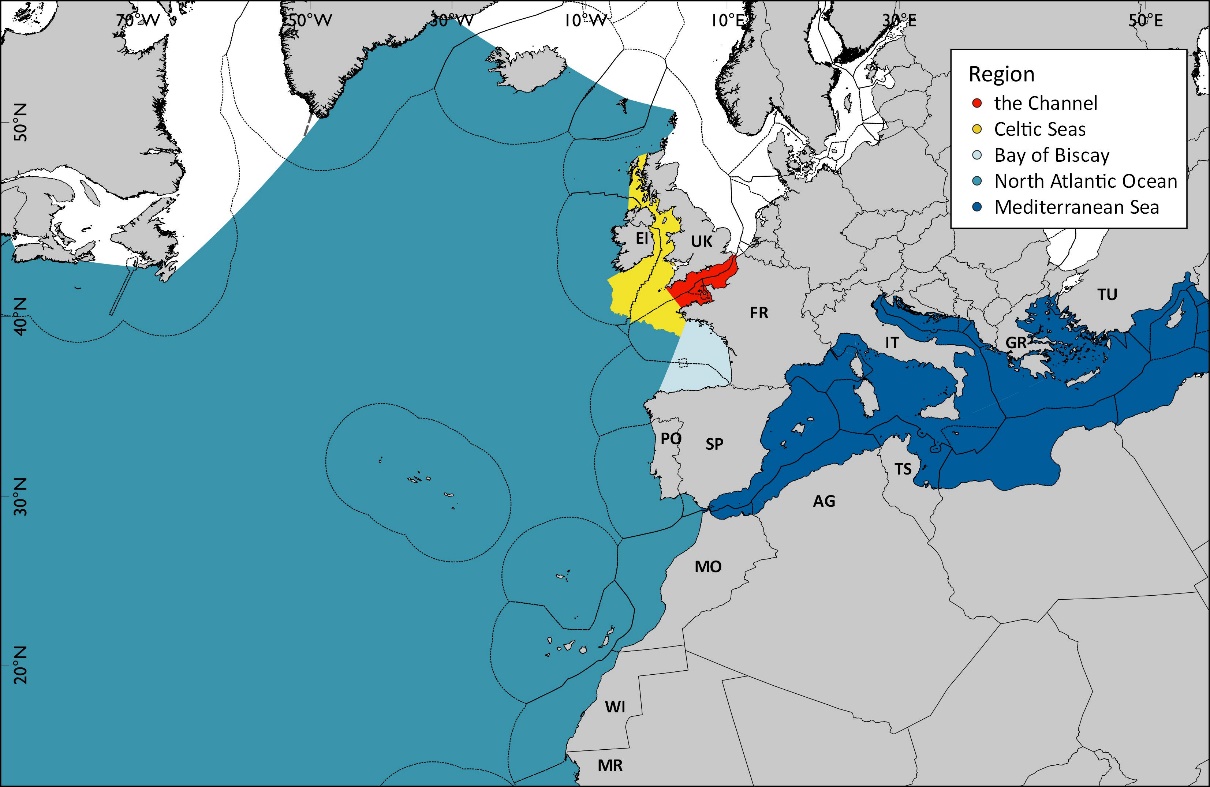
**

**Figure S3. Spatial regions used in this study.** Exclusive Economic Zones (EEZ) are shown as outlined polygons and International Hydrographic Organization (IHO) regions as filled polygons.

**
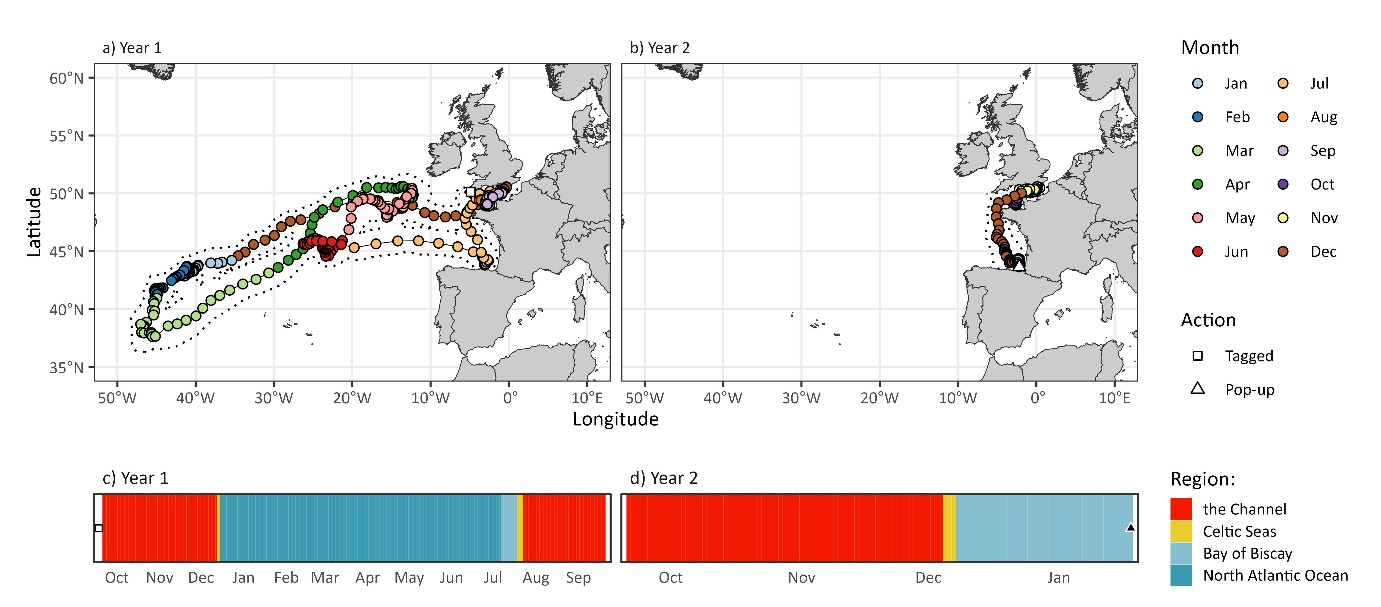
**

**Figure S4. Reconstructed movements for 20P0094 (190 cm at the time of tagging) over 481 tracking days.** a-b) Modelled daily locations for each tracking year. Area outlined by dotted line denotes the 99% probability surface. c-d) Residency over time summarised by IHO region occupied


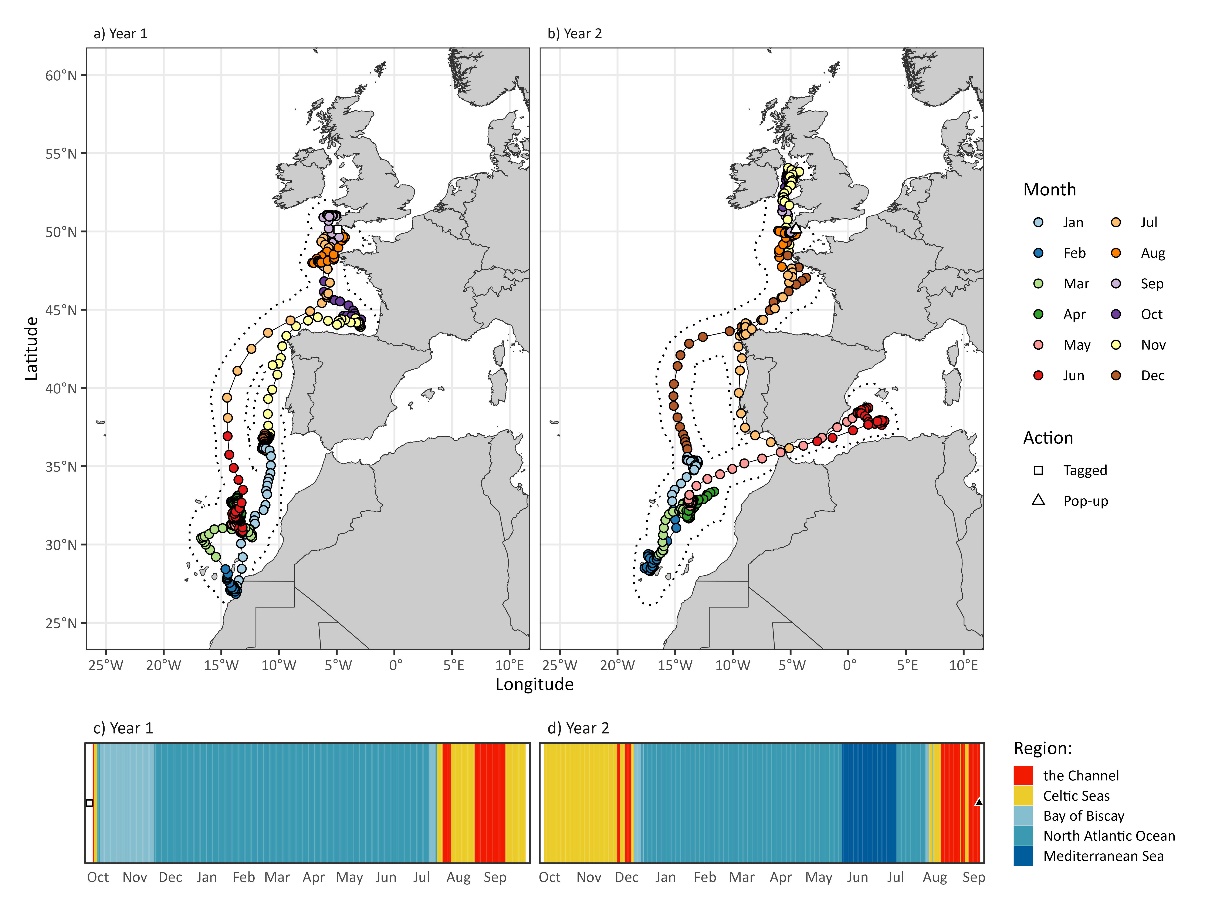
**Figure S5. Reconstructed movements for 20P0056 (241 cm at the time of tagging) over 709 tracking days.** a-b) Modelled daily locations for each tracking year. Area outlined by dotted line denotes the 99% probability surface. c-d) Residency over time summarised by IHO region occupied

|  |  | Period | | |
| --- | --- | --- | --- | --- |
| IHO | **EEZ** | **On-shelf (Aug-Nov)** | **Off-shelf (Dec-Apr)** | **Spawn (May-Jul)** |
| The Channel | United Kingdom | 0.32 ± 0.16 (0.06-0.6) | 0.13 ± 0.09 (0.01-0.27) | 0.05 ± 0.03 (0.01-0.08) |
|  | France | 0.23 ± 0.14 (0.02-0.48) | 0.08 ± 0.06 (0.02-0.16) | 0.04 ± 0.03 (0.01-0.08) |
| Celtic Seas | Ireland | 0.08 ± 0.07 (0.01-0.2) | 0.07 ± 0.08 (0.01-0.23) | 0.03 ± 0.02 (0.01-0.08) |
|  | United Kingdom | 0.17 ± 0.12 (0.01-0.39) | 0.05 ± 0.04 (0.01-0.14) | 0.05 ± 0.01 (0.03-0.08) |
|  | France | 0.09 ± 0.07 (0.01-0.27) | 0.11 ± 0.08 (0.02-0.28) | 0.04 ± 0.03 (0.01-0.14) |
| Bay of Biscay | France | 0.19 ± 0.14 (0.02-0.41) | 0.27 ± 0.19 (0.02-0.78) | 0.21 ± 0.2 (0.02-0.71) |
|  | Spain | 0.18 ± 0.09 (0.07-0.35) | 0.12 ± 0.12 (0.01-0.51) | 0.22 ± 0.19 (0.01-0.63) |
| North Atlantic Ocean | Ireland | 0.13 ± 0.09 (0.04-0.21) | 0.11 ± 0.12 (0.02-0.3) | 0.09 ± 0.07 (0.01-0.23) |
|  | United Kingdom | - | 0.05 ± 0.04 (0.01-0.13) | 0.01 ± 0 (0.01-0.02) |
|  | France | 0.05 ± 0.04 (0.01-0.12) | 0.15 ± 0.15 (0.02-0.46) | 0.05 ± 0.03 (0.01-0.11) |
|  | Spain | 0.09 ± 0.1 (0.02-0.16) | 0.05 ± 0.03 (0.01-0.11) | 0.1 ± 0.05 (0.02-0.23) |
|  | Portugal | - | 0.18 ± 0.22 (0.01-0.64) | 0.13 ± 0.08 (0.07-0.31) |
|  | Morocco | - | - | 0.04 ± 0.01 (0.03-0.05) |
|  | High Seas | 0.07 ± 0.09 (0.01-0.13) | 0.34 ± 0.21 (0.02-0.77) | 0.21 ± 0.22 (0.01-0.6) |
| Mediterranean Sea | Morocco | - | - | 0.01 ± 0 (0.01-0.02) |
|  | Spain | - | - | 0.19 ± 0.19 (0.05-0.61) |
|  | Algeria | - | - | 0.13 ± 0.07 (0.04-0.26) |
|  | Tunisia | - | - | 0.12 ± 0.03 (0.08-0.16) |
|  | Malta | - | - | 0.04 ± 0.01 (0.03-0.04) |
|  | Italy | - | - | 0.13 ± 0.11 (0.01-0.26) |
|  | Libya | - | - | 0.16 ± 0.11 (0.01-0.29) |

**Table S2. Summary of mean probability of occurrence of Atlantic bluefin tuna by both exclusive economic zone (EEZ) and International Hydrographic Organisation (IHO) region.** Shading denotes the two most likely national/IHO combinations for each period, with the darkest shading being the most probable. Dashes denote one or less fish tracked.

**Table S3. Summary of percentage time Atlantic bluefin tuna spent in both exclusive economic zone (EEZ) and International Hydrographic Organisation (IHO) regions.** Shading denotes the two most occupied national/IHO combinations for each period, with the darkest shading being the most occupied. Dashes denote one or less fish tracked.

|  |  | Period | | |
| --- | --- | --- | --- | --- |
| IHO | **EEZ** | **On-shelf (Aug-Nov)** | **Off-shelf (Dec-Apr)** | **Spawn (May-Jul)** |
| The Channel | United Kingdom | 29 ± 19% (1-65) | 14 ± 9% (1-28) | 5 ± 3% (1-9) |
|  | France | 24 ± 16% (1-54) | 7 ± 6% (1-16) | 5 ± 3% (1-10) |
| Celtic Seas | Ireland | 8 ± 6% (2-16) | 7 ± 8% (1-23) | 3 ± 3% (1-9) |
|  | United Kingdom | 19 ± 12% (1-38) | 5 ± 6% (0-16) | 5 ± 2% (1-8) |
|  | France | 9 ± 8% (1-26) | 11 ± 8% (0-30) | 4 ± 4% (1-14) |
| Bay of Biscay | France | 18 ± 14% (2-41) | 27 ± 21% (1-84) | 21 ± 23% (1-82) |
|  | Spain | 18 ± 10% (1-34) | 13 ± 15% (0-56) | 25 ± 20% (1-66) |
| North Atlantic Ocean | Ireland | 13 ± 7% (7-21) | 12 ± 13% (2-36) | 9 ± 7% (1-24) |
|  | United Kingdom | 59% (59) | 4 ± 5% (1-13) | 2 ± 1% (1-3) |
|  | France | 4 ± 4% (1-12) | 14 ± 16% (0-51) | 5 ± 3% (1-12) |
|  | Spain | 10 ± 10% (2-17) | 5 ± 3% (1-11) | 9 ± 5% (1-24) |
|  | Portugal | 4% (4) | 16 ± 23% (1-68) | 14 ± 8% (7-31) |
|  | Morocco | - | 6% (6) | 4 ± 2% (2-5) |
|  | High Seas | 13% (13) | 36 ± 22% (1-79) | 20 ± 22% (1-61) |
| Mediterranean Sea | Morocco | - | - | 1% (1) |
|  | Spain | - | - | 18 ± 20% (4-63) |
|  | Algeria | - | - | 14 ± 7% (4-28) |
|  | Tunisia | - | - | 12 ± 5% (5-18) |
|  | Malta | - | - | 2 ± 1% (1-2) |
|  | Italy | - | - | 18 ± 12% (1-31) |
|  | Libya | - | - | 22 ± 8% (15-30) |

**Table S4. Summary of days Atlantic bluefin tuna spent in both exclusive economic zone (EEZ) and International Hydrographic Organisation (IHO) regions.** Shading denotes the two most occupied national/IHO combinations for each period, with the darkest shading being the most occupied. Dashes denote one or less fish tracked.

|  |  | Period | | |
| --- | --- | --- | --- | --- |
| IHO | **EEZ** | **On-shelf (Aug-Nov)** | **Off-shelf (Dec-Apr)** | **Spawn (May-Jul)** |
| The Channel | United Kingdom | 35 ± 25 d (1-88) | 22 ± 13 d (3-42) | 5 ± 3 d (1-8) |
|  | France | 28 ± 25 d (1-105) | 11 ± 10 d (1-24) | 5 ± 3 d (1-9) |
| Celtic Seas | Ireland | 9 ± 6 d (2-20) | 11 ± 12 d (2-35) | 3 ± 2 d (1-8) |
|  | United Kingdom | 23 ± 19 d (1-75) | 8 ± 8 d (1-24) | 5 ± 2 d (1-7) |
|  | France | 10 ± 9 d (1-32) | 16 ± 13 d (1-46) | 4 ± 3 d (1-13) |
| Bay of Biscay | France | 21 ± 17 d (2-50) | 41 ± 31 d (1-128) | 20 ± 21 d (1-75) |
|  | Spain | 19 ± 12 d (1-42) | 21 ± 22 d (1-85) | 22 ± 18 d (1-61) |
| North Atlantic Ocean | Ireland | 16 ± 9 d (6-26) | 19 ± 20 d (3-54) | 8 ± 7 d (1-22) |
|  | United Kingdom | 71 d (71) | 7 ± 7 d (1-20) | 1 ± 1 d (1-3) |
|  | France | 5 ± 5 d (1-15) | 21 ± 24 d (1-77) | 4 ± 3 d (1-11) |
|  | Spain | 12 ± 11 d (5-20) | 7 ± 5 d (1-16) | 9 ± 5 d (1-22) |
|  | Portugal | - | 31 ± 52 d (1-162) | 13 ± 12 d (6-53) |
|  | Morocco |  | 17 d (17) | 4 ± 1 d (3-4) |
|  | High Seas | 15 d (15) | 59 ± 37 d (1-120) | 19 ± 20 d (1-56) |
| Mediterranean Sea | Morocco | - | - | 1 d (1) |
|  | Spain | - | - | 17 ± 18 d (4-58) |
|  | Algeria | - | - | 12 ± 6 d (4-26) |
|  | Tunisia | - | - | 10 ± 5 d (4-16) |
|  | Malta | - | - | 1 ± 0 d (1-1) |
|  | Italy | - | - | 15 ± 11 d (1-28) |
|  | Libya | - | - | 20 ± 7 d (14-28) |

**
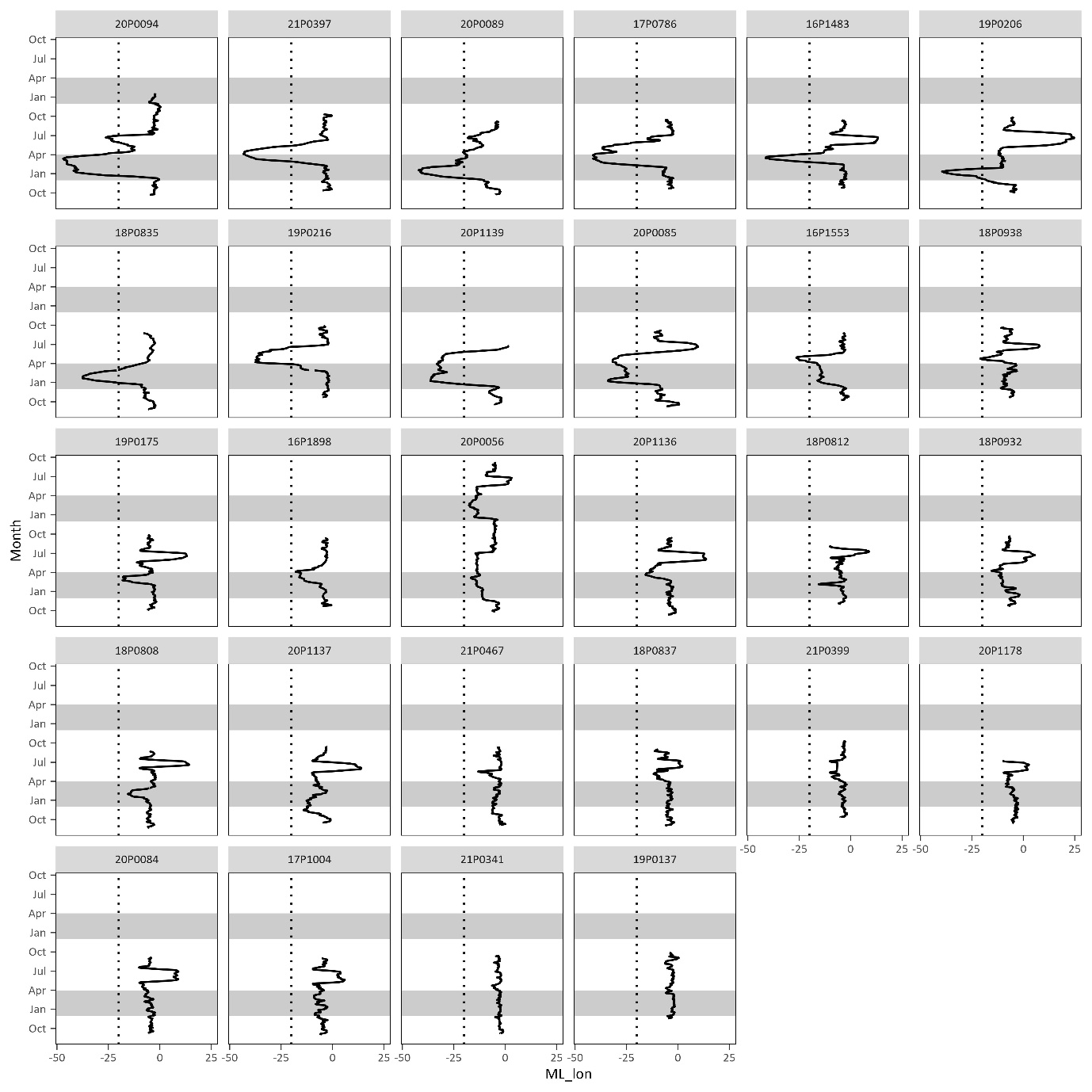
Figure S6. Longitudinal movements of tracked ABT**

**
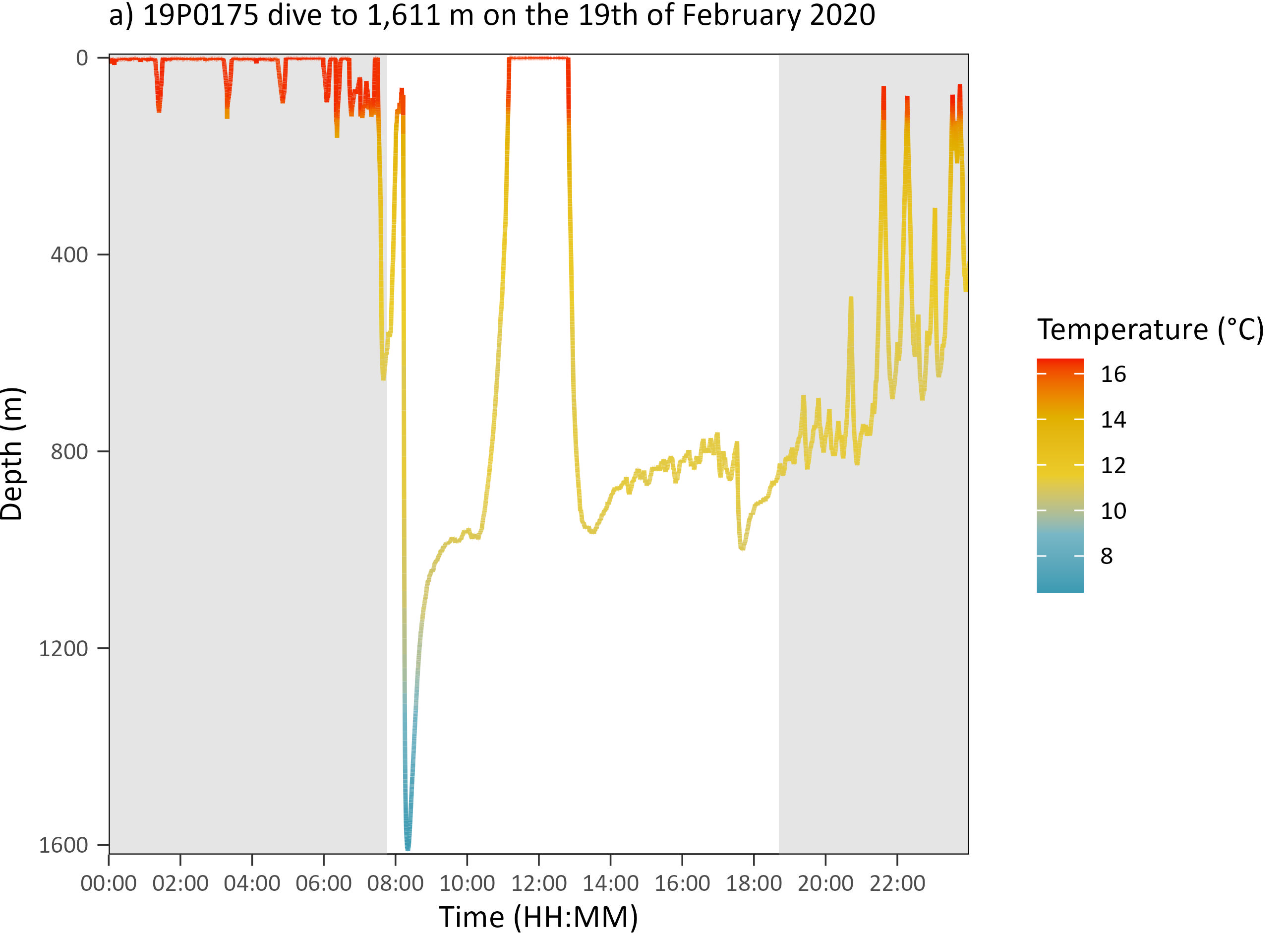
**

**Figure S7. The deepest dive made by an Atlantic bluefin tuna in this study.** Grey rectangles denote nighttime periods as devised from sensed light levels.

**
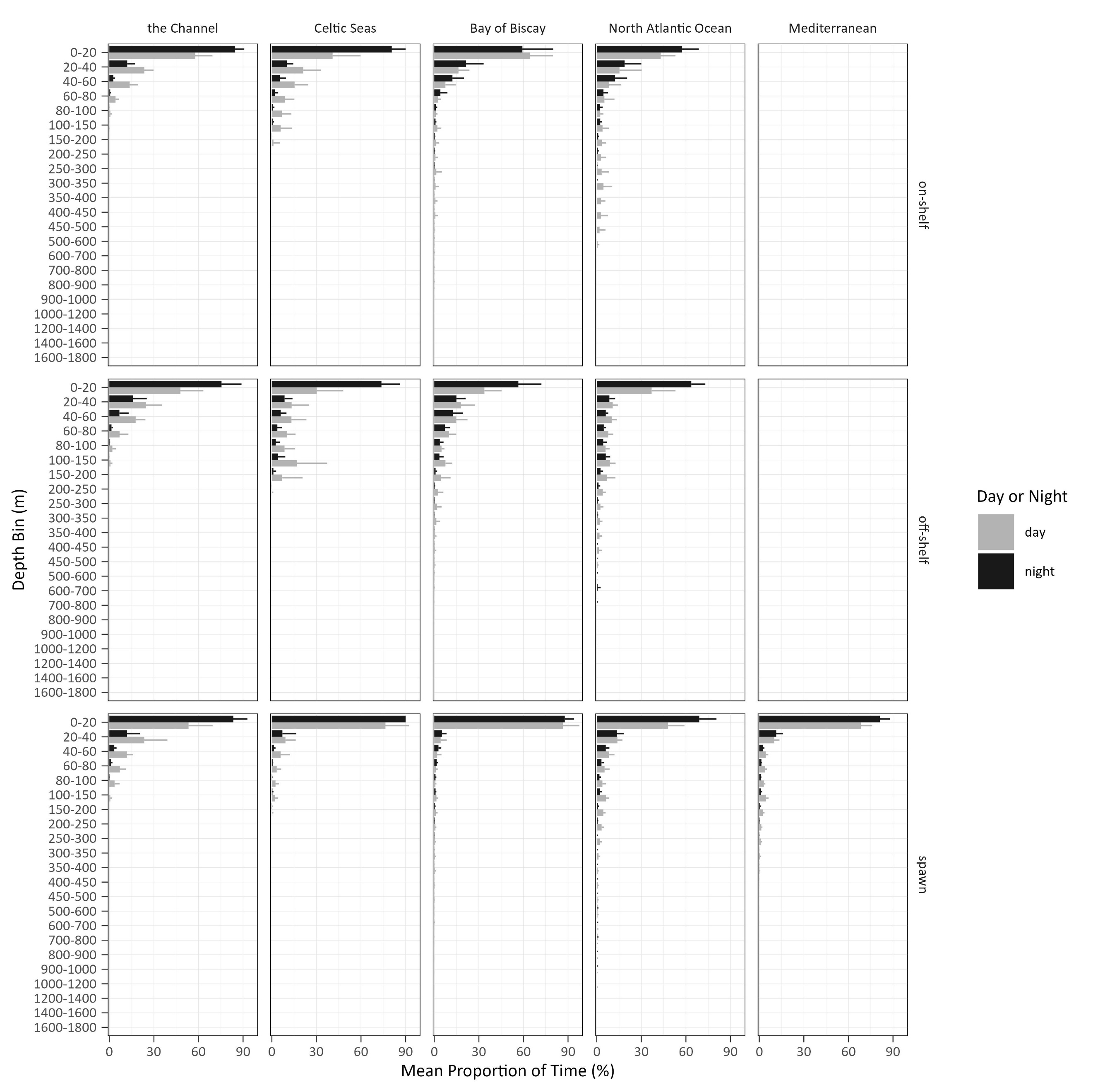
**

**Figure S8. Proportion of time spent at different depths by day and nighttime period, geographic region and tracking phase.** Error bars denote ± 1. Standard Error. Sample size is the 19 tags (i.e. tags that were attached for >= 300 days and had functioning depth sensors).

**
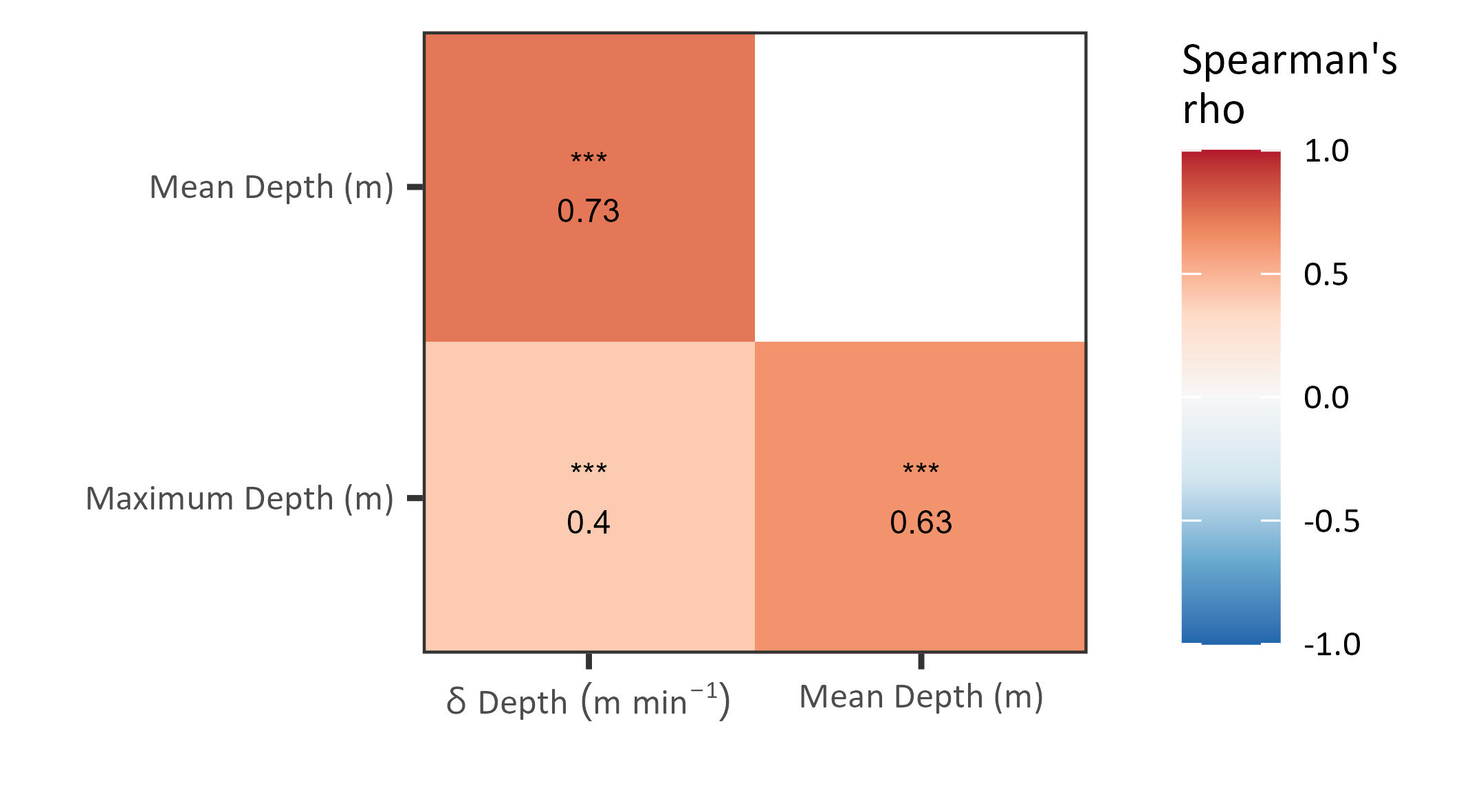
**

**Figure S9. Correlation matrix for vertical habitat use metrics.** Metrics were tested by applying Spearman’s rank order correlation. Significance at the P = <0.001 level is denoted by the symbol “***” and cells are shaded according to the test statistic obtained (Spearman’s rho).

**Table S5.** Generalised Linear Mixed Model specifications and results of a comparing reduced models with the full models by conducting a likelihood ratio test. P (<0.05) refers to the values obtained when conducting a Ljung Box Test on standardized model residuals using the *Box.test* function in the “statistics” package in R. “DWT” denotes the Durbin-Watson test statistic calculated using standardized model residuals and the the *durbinWatsonTest* function in the “car” package in R.

| Response | | Auto-regressive structure | | Num df | AICc | Δ AICc | P (<0.05) | DWT |
| --- | --- | --- | --- | --- | --- | --- | --- | --- |
| Mean Depth Occupied (m) | corARMA(2,2,~1) | | | 25 | 25548 | 0 | 0.28 | 1.98 |
|  | corARMA(3,0,~1) | | | 24 | 26449 | 921 | 0.01 | 1.95 |
|  | corARMA(2,0,~1) | | | 23 | 26,617 | 168 | <0.001 | 1.88 |
|  | corARMA(1,0,~1) | | | 22 | 30,083 | 3,634 | <0.001 | 2.18 |
|  | None | | | 21 | 30,454 | 4,004 | <0.001 | 1.65 |
| *Final model:* | | | *log(Mean Depth Occupied) ~ Daytime/Nighttime * Moon illumination + Horizontal displacement (km.d^-1^) + Tracking phase*Region, random = ~1\|TagID, correlation = corARMA(2,2,~1)* | | | | | |
| Maximum Depth (m) | corARMA(3,0,~1) | | | 24 | 17,216 | 0 | 0.43 | 1.99 |
|  | corARMA(2,0,~1) | | | 23 | 17,237 | 20 | 0.09 | 1.97 |
|  | corARMA(1,0,~1) | | | 22 | 19,044 | 1,827 | <0.001 | 2.23 |
|  | None | | | 21 | 20,155 | 2,938 | <0.001 | 1.4 |
| *Final model:* | *log(Maximum Depth) ~ Daytime/Nighttime + Moon illumination + Horizontal displacement (km.d^-1^) + Tracking phase*Region + random = ~1\|TagID, correlation = corARMA(3,0,~1)* | | | | | | | |
| Vertical Movement Rate (m.min^-1^) | corARMA(2,3,~1) | | | 26 | 14532 | 0 | 0.48 | 1.99 |
|  | corARMA(2,2,~1) | | | 25 | 14584 | 170 | 0.03 | 1.96 |
|  | corARMA(3,0,~1) | | | 24 | 15511 | 1097 | <0.001 | 1.95 |
|  | corARMA(2,0,~1) | | | 23 | 15,684 | 173 | <0.001 | 1.85 |
|  | corARMA(1,0,~1) | | | 22 | 21,282 | 5,771 | <0.001 | 2.11 |
|  | None | | | 21 | 21,372 | 5,861 | <0.001 | 1.82 |
| *Final model:* | *sqrt(Vertical Movement Rate) ~ Daytime/Nighttime * Moon illumination + Horizontal displacement (km.d^-1^) + Tracking phase*Region, random = ~1\|TagID, correlation = corARMA(2,3,~1)* | | | | | | | |

**Table S6.** Generalised Linear Mixed Model specifications and results of a comparing reduced models with the full models by conducting a Chi squared test (Χ_2_).

| Response | | Fixed effect | Num df | Χ_2_ | Pr (>Χ_2_) |
| --- | --- | --- | --- | --- | --- |
| Mean Depth Occupied (m) | Horizontal displacement (km.d^-1^) | | 1 | 16.2 | <0.001*** |
|  | Daytime/Nighttime * Moon illumination | | 2 | 77.8 | <0.001*** |
|  | Tracking phase*Region | | 12 | 289.8 | <0.001*** |
| Maximum Depth (m) | Horizontal displacement (km.d^-1^) | | 1 | 0.6 | 0.44 |
|  | Daytime/Nighttime | | 1 | 71.4 | <0.001*** |
|  | Moon illumination | | 2 | 0.2 | 0.89 |
|  | Tracking phase*Region | | 12 | 850.9 | <0.001*** |
| Vertical Movement Rate (m min^-1^) | Horizontal displacement (km.d^-1^) | | 1 | 22.1 | <0.001*** |
|  | Daytime/Nighttime * Moon illumination | | 2 | 21.6 | <0.001*** |
|  | Tracking phase*Region | | 12 | 93 | <0.001*** |

|  |  | | Tracking phase | |  | |  |
| --- | --- | --- | --- | --- | --- | --- | --- |
|  | **On-shelf** | | **Off-shelf** | | **Spawn** | | **Grand mean (region)** |
| Region | **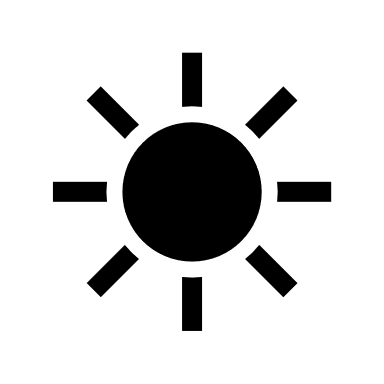** | **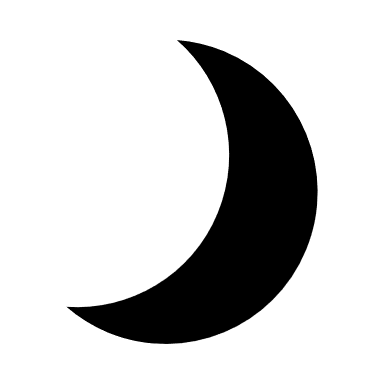** | **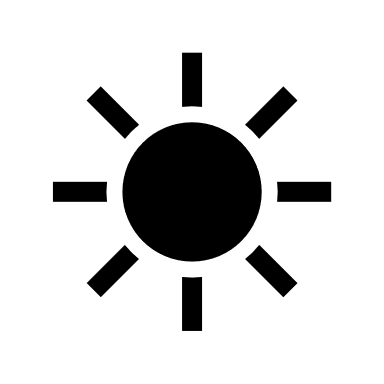** | **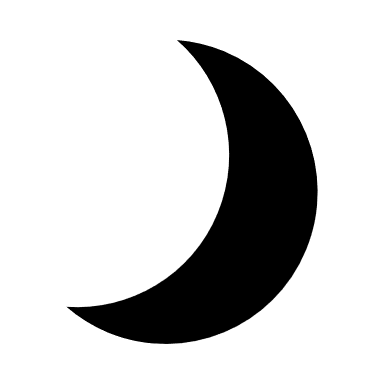** | **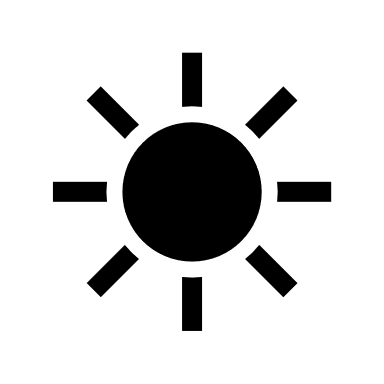** | **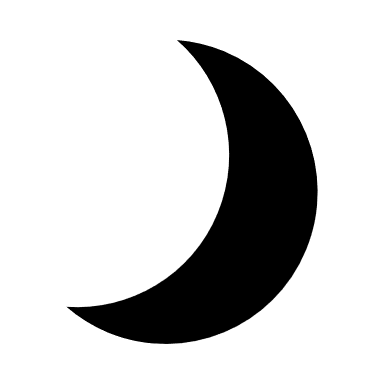** |  |
| *Mean Depth Occupied (m)* | | | | | | | |
| the Channel | 22 ± 5 | 11 ± 3 | 27 ± 7 | 14 ± 5 | 25 ± 4 | 11 ± 3 | 18 ± 7 |
| Celtic Seas | 38 ± 18 | 13 ± 5 | 60 ± 29 | 21 ± 11 | 18 ± 9 | 8 ± 3 | 26 ± 19 |
| Bay of Biscay | 36 ± 32 | 23 ± 9 | 63 ± 34 | 29 ± 10 | 21 ± 11 | 13 ± 4 | 31 ± 18 |
| North Atlantic Ocean | 91 ± 63 | 28 ± 5 | 84 ± 29 | 44 ± 28 | 72 ± 27 | 36 ± 30 | 59 ± 27 |
| Mediterranean Sea | - | - | - | - | 32 ± 10 | 16 ± 4 | 24 ± 11 |
| Grand mean (day/night) | 47 ± 30 | 19 ± 8 | 59 ± 24 | 27 ± 13 | 34 ± 22 | 17 ± 11 |  |
| Grand mean (phase) | 33 ± 25 | | 43 ± 24 | | 25 ± 19 | |  |
| *Maximum Depth Reached (m)* | | | | | | | |
| the Channel | 68 ± 6 | 63 ± 7 | 75 ± 14 | 78 ± 11 | 86 ± 15 | 76 ± 13 | 74 ± 8 |
| Celtic Seas | 105 ± 25 | 98 ± 33 | 135 ± 33 | 138 ± 34 | 117 ± 27 | 87 ± 23 | 113 ± 20 |
| Bay of Biscay | 160 ± 89 | 164 ± 49 | 186 ± 70 | 200 ± 55 | 194 ± 60 | 142 ± 39 | 174 ± 23 |
| North Atlantic Ocean | 313 ± 105 | 287 ± 85 | 300 ± 74 | 296 ± 120 | 325 ± 77 | 243 ± 114 | 294 ± 28 |
| Mediterranean Sea | - | - | - | - | 220 ± 47 | 150 ± 29 | 185 ± 50 |
| Grand mean (day/night) | 162 ± 108 | 153 ± 99 | 174 ± 96 | 178 ± 93 | 188 ± 94 | 140 ± 66 |  |
| Grand mean (phase) | 157 ± 96 | | 176 ± 87 | | 164 ± 81 | |  |
| *Vertical Movement Rate (m.min^-1^)* | | | | | | | |
| the Channel | 10 ± 1.8 | 6.2 ± 0.8 | 10.6 ± 2.5 | 6.4 ± 1.3 | 11.7 ± 2.6 | 7.6 ± 1.7 | 8.8 ± 2.3 |
| Celtic Seas | 12.3 ± 2.7 | 6.6 ± 1 | 12.7 ± 3.7 | 6.6 ± 1.2 | 10.9 ± 2.1 | 5.9 ± 1.2 | 9.2 ± 3.1 |
| Bay of Biscay | 8.4 ± 2.4 | 6.4 ± 0.7 | 10.3 ± 2.1 | 6.7 ± 1.2 | 7.5 ± 2.8 | 6.6 ± 0.5 | 7.7 ± 1.5 |
| North Atlantic Ocean | 12.2 ± 2.4 | 8.5 ± 2.5 | 13.6 ± 3.9 | 7.4 ± 1.4 | 14.8 ± 2.9 | 8.8 ± 2.4 | 10.9 ± 3.1 |
| Mediterranean Sea | - | - | - | - | 8.9 ± 1.3 | 7.3 ± 1 | 8.1 ± 1.1 |
| Grand mean (day/night) | 10.7 ± 1.9 | 6.9 ± 1.1 | 11.8 ± 1.6 | 6.8 ± 0.4 | 10.8 ± 2.8 | 7.2 ± 1.1 |  |
| Grand mean (phase) | 8.8 ± 2.5 | | 9.3 ± 2.9 | | 9 ± 2.7 | |  |

**Table S7. Summary statistics for diving behaviour determined from 19 recovered MiniPAT archives.**

**
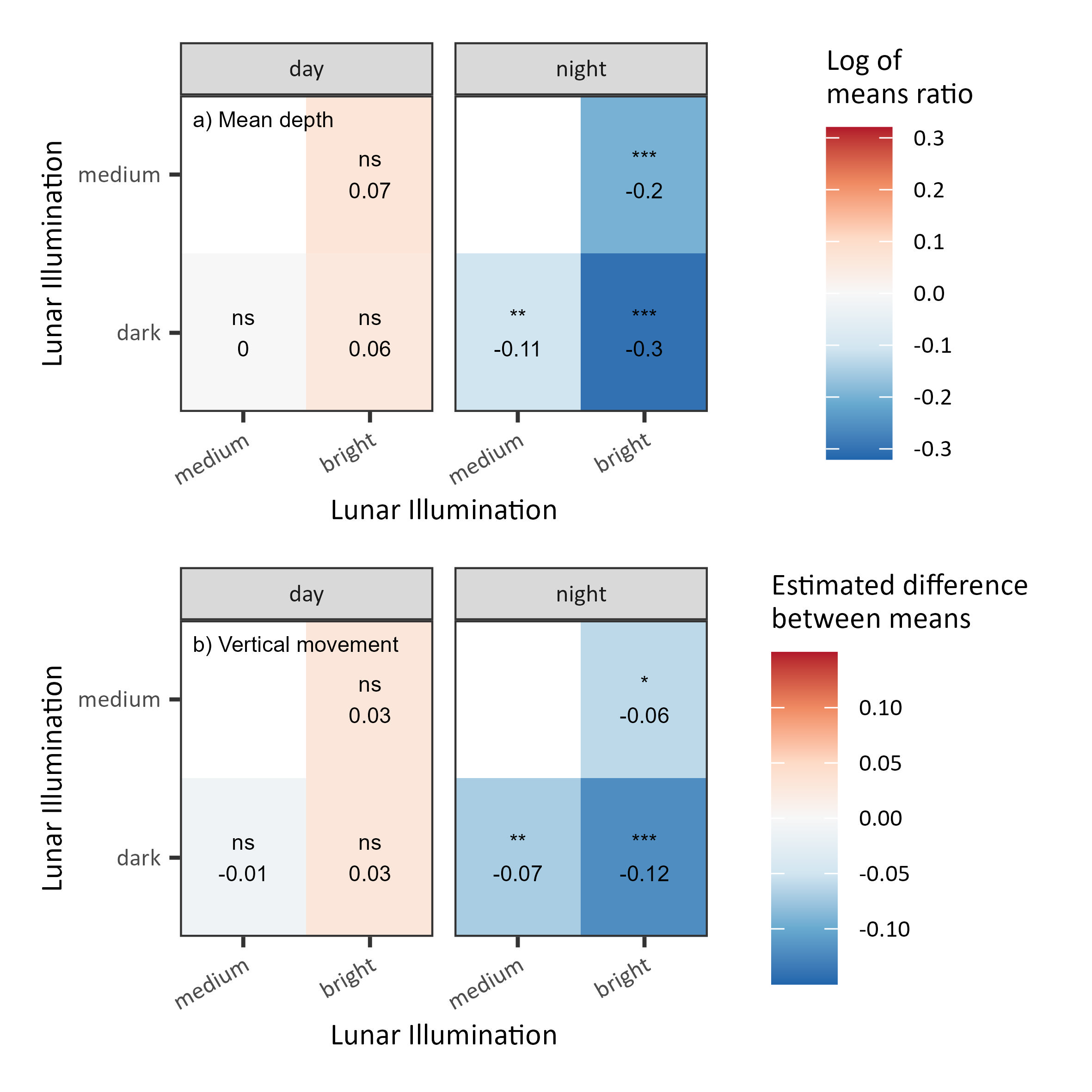
**

**Figure S10. Correlation matrix for interaction of daytime or nighttime and lunar illumination identified in GLMM.** Significance is denoted using symbols as follows = “ns”, not significant at the P = <0.05 level, “**” significant at the P = <0.01 level, and “***” significant at the P = <0.001 level. Boxes are shaded according to the comparison of means, which for log transformed data (mean depth) is expressed as the log of the ratio of geometric means, and for square root transformed data as the difference between modelled means. Maximum depth is not shown as the results were non-significant.


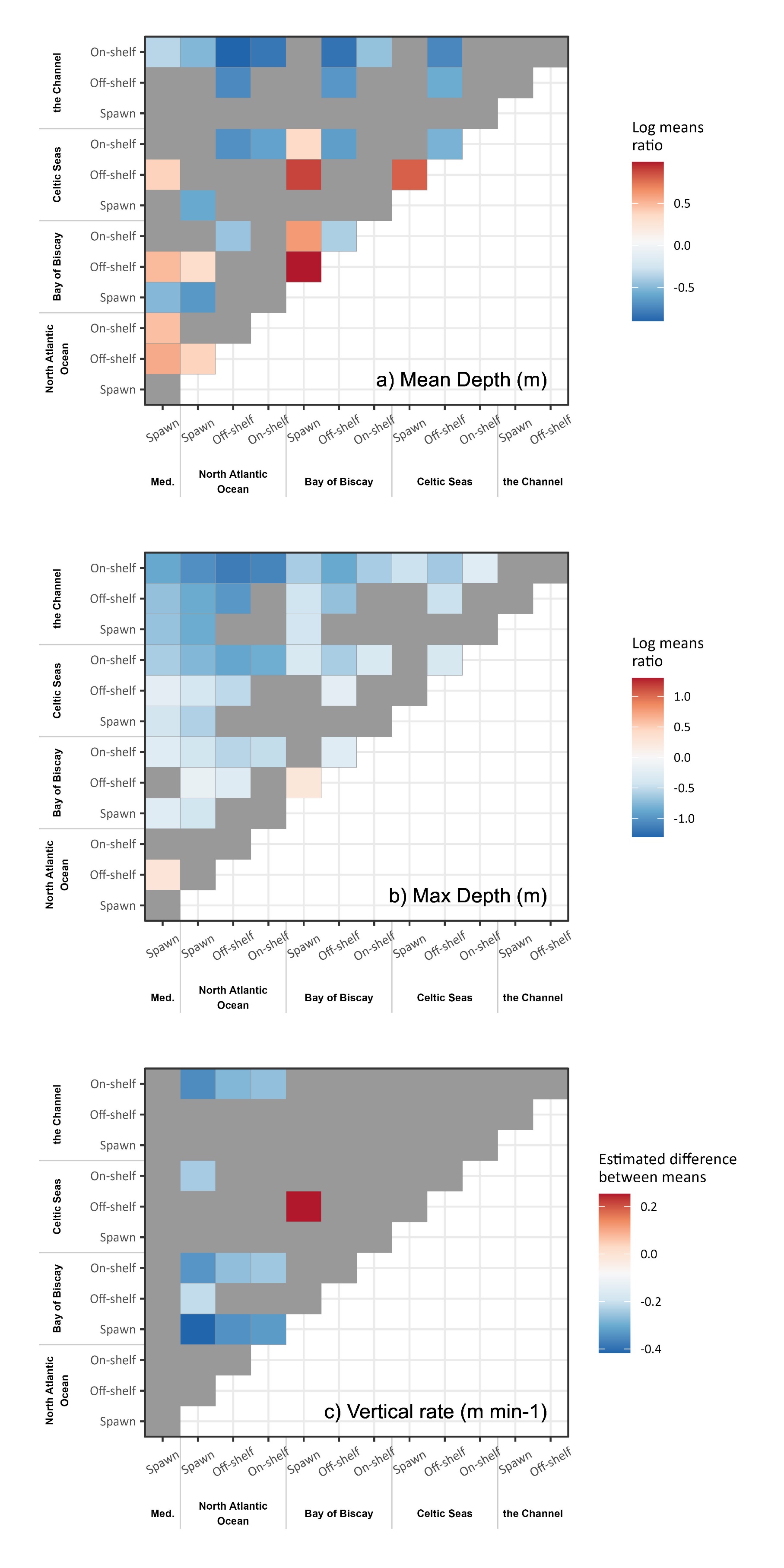


**Figure S11. Correlation matrix for interaction of region and tracking phase identified in GLMM.** Grey shaded boxes denote no significant effect at the P = <0.05 level. Boxes are shaded according to the comparison of means, which for log transformed data (mean depth and maximum depth) is expressed as the log of the ratio of geometric means, and for square root transformed data (Vertical rate) as the difference between modelled means.

**
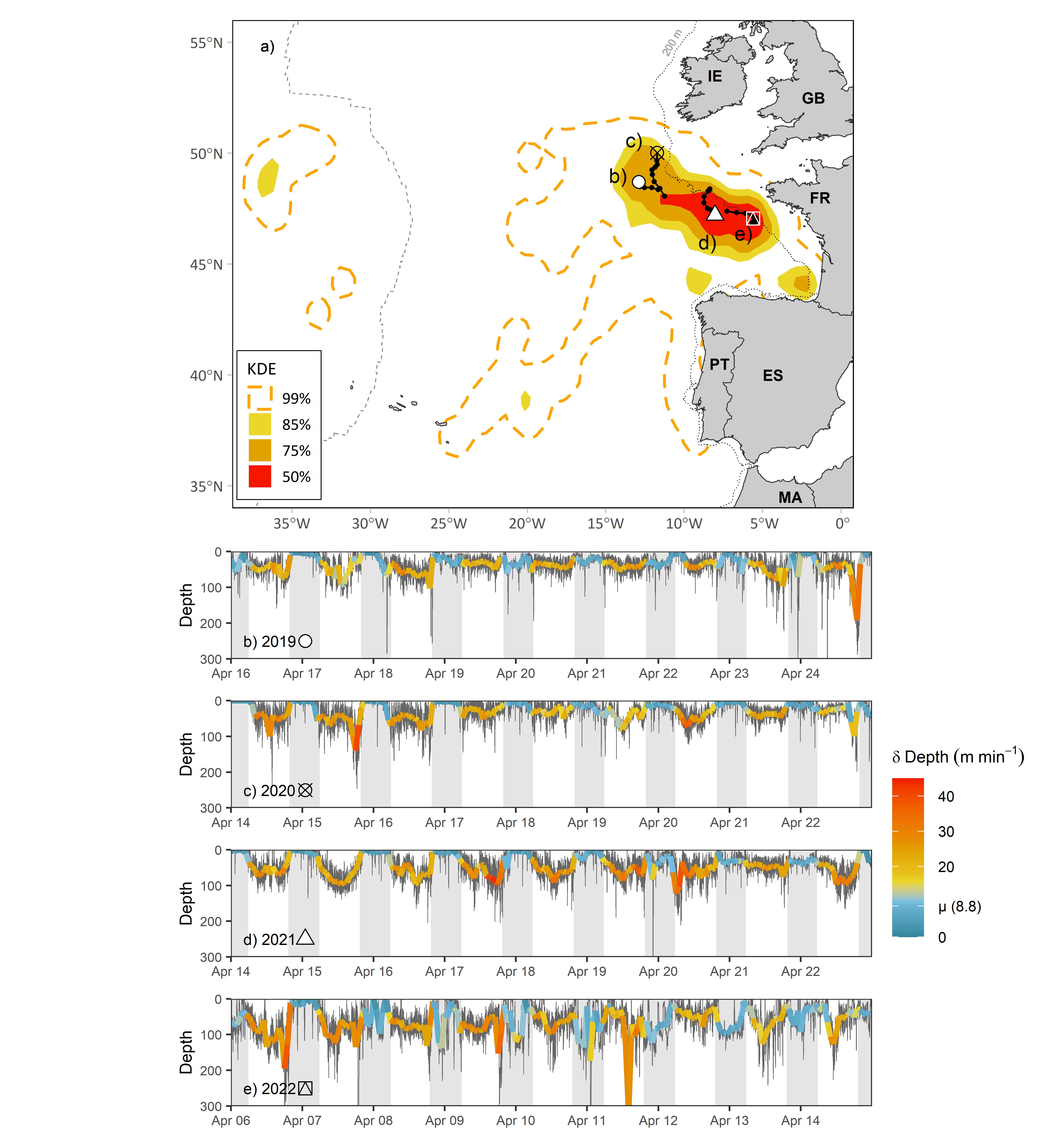
**

**Figure S12. Localised, oscillatory diving behaviour of Atlantic bluefin tuna revealed from archival tags.** a) Map of the northeast Atlantic showing utilisation distribution (UD) kernels with a 2° smoothing parameter of daily modelled locations for 19 tags where shallow, oscillatory behaviour was observed (characterised as >P_90_ of daily mean δ depth). 9-day tracks of four example fish, one for each of the 2019, 2020, 2021 and 2022 tracking years, are overlaid and correspond to time series in adjacent plots. b-e) Time series of raw diving behaviour (grey line) with 10-minutely summed mean depth overlaid and coloured by depth change. The grand mean daily diving rate (µ) is indicated on the colour scale for reference.
